# Predicting the Adsorption of Amoxicillin and Ibuprofen on Chitosan and Graphene Oxide Materials: A Density Functional Theory Study

**DOI:** 10.3390/polym13101620

**Published:** 2021-05-17

**Authors:** Leonardo Anchique, Jackson J. Alcázar, Andrea Ramos-Hernandez, Maximiliano Méndez-López, José R. Mora, Norma Rangel, José Luis Paz, Edgar Márquez

**Affiliations:** 1 Programa de Química, Grupo Química Supramolecular Aplicada, Facultad de Ciencias Básicas, Semillero Electroquímica Aplicada, Universidad del Atlántico, Barranquilla 081001, Colombia; lanchique@mail.uniatlantico.edu.co (L.A.); andrearamos@mail.uniatlantico.edu.co (A.R.-H.); 2 Facultad de Química y de Farmacia, Pontificia Universidad Católica de Chile, Casilla 306, Santiago 6094411, Chile; jjalcazar@uc.cl; 3 Departamento de Química y Biología, Facultad de Ciencias Exactas, Grupo de Investigaciones en Química y Biología, Universidad del Norte, Carrera 51B, Km 5, vía Puerto Colombia, Barranquilla 081007, Colombia; 4 Departamento de Ingeniería Química, Grupo de Química Computacional y Teórica (QCT-USFQ), Diego de Robles y Vía Interoceánica, Universidad San Francisco de Quito, Quito 170901, Ecuador; 5 TecNM/Instituto Tecnológico de Aguascalientes-División de Estudios de Posgrado e Investigación, Ave. Adolfo López Mateos #1801Ote. Fracc. Bona Gens, Aguascalientes 20256, Mexico; norma.rv@aguascalientes.tecnm.mx; 6 Departamento Académico de Química Inorgánica, Facultad de Química e Ingeniería Química, Universidad Nacional Mayor de San Marcos, Cercado de Lima 15081, Peru; jpazr@unmsm.edu.pe

**Keywords:** emergent pollutants, pharmaceuticals, absorption, density functional theory, natural bond orbital

## Abstract

The occurrence, persistence, and accumulation of antibiotics and non-steroidal anti-inflammatory drugs (NSAIDs) represent a new environmental problem due to their harmful effects on human and aquatic life. A suitable absorbent for a particular type of pollutant does not necessarily absorb other types of compounds, so knowing the compatibility between a particular pollutant and a potential absorbent before experimentation seems to be fundamental. In this work, the molecular interactions between some pharmaceuticals (amoxicillin, ibuprofen, and tetracycline derivatives) with two potential absorbers, chitosan and graphene oxide models (pyrene, GO-1, and coronene, GO-2), were studied using the ωB97X-D/6-311G(2d,p) level of theory. The energetic interaction order found was amoxicillin/chitosan > amoxicillin/GO-1 > amoxicillin/GO-2 > ibuprofen/chitosan > ibuprofen/GO-2 > ibuprofen/GO-1, the negative sign for the interaction energy in all complex formations confirms good compatibility, while the size of E_int_ between 24–34 kcal/mol indicates physisorption processes. Moreover, the free energies of complex formation were negative, confirming the spontaneity of the processes. The larger interaction of amoxicillin Gos, compared to ibuprofen Gos, is consistent with previously reported experimental results, demonstrating the exceptional predictability of these methods. The second-order perturbation theory analysis shows that the amoxicillin complexes are mainly driven by hydrogen bonds, while van der Waals interactions with chitosan and hydrophobic interactions with graphene oxides are modelled for the ibuprofen complexes. Energy decomposition analysis (EDA) shows that electrostatic energy is a major contributor to the stabilization energy in all cases. The results obtained in this work promote the use of graphene oxides and chitosan as potential adsorbents for the removal of these emerging pollutants from water.

## 1. Introduction

The greatest challenge facing humanity today is the preservation of the Earth as a habitat [1,2,3]. The challenge becomes even more dramatic when one considers the exponential growth of the world’s population, which demands not only more clean water sources but also modern water treatment methods [4].

Freshwater supplies are threatened by the increase in pharmaceutical pollutants, with around 70% coming from domestic water and 30% from industrial residues, which are discharged directly into rivers without prior treatment [5,6].

Although pharmaceuticals are strictly regulated for patient safety, the side effect on the environment is not yet regulated. Moreover, the side effect of any type of drug (antibiotics, analgesics, antipyretics, etc.) on the environment is not well known. However, it is already known that they continuously enter the environment day after day through wastewater. At the same time, most pharmaceutical pollutants have a considerable prevalence in low concentrations; therefore, the risk of this accumulation in critical concentrations affecting life, especially in the aquatic environment, seems probable [7,8,9].

In the last five years, several papers have been reported dealing with the fate and presence of pharmaceutical contaminants in fresh and drinking water sources [10,11,12,13]. In most of them, the presence of both antibiotics and non-steroidal anti-inflammatory drugs (NSAID) is a critical health situation. Antibiotics have become of great concern not only because of their prolonged persistence in the environment but also because of the risk of emergence of antibiotic resistance as new pollutants. Moreover, some authors point out the potential toxicity, genotoxicity, and teratogenicity to aquatic organisms [14,15,16,17]. On the other hand, NSAIDs are known as the drugs whose occurrence and prevalence have been reported the most [18,19,20]. Both the structural diversity and stability of these types of pharmaceutical determine their persistence in the environment. In addition to their persistence and various acute toxicities, some authors have reported the decline of the vulture population in Switzerland, India, Pakistan, Bangladesh, and Nepal as a result of poisoning with NSAIDS [21,22,23]. High toxicity to plants and microorganisms has also been reported [24,25,26].

Both of the aforementioned types of compounds can be obtained inexpensively, mostly in third world countries; therefore, easy access to drugs without optional prescriptions makes the accumulation of these compounds inevitable in a short time. Considering that the usual water treatment methods such as oxidation, Fenton reaction, filtration, biological treatment, and precipitation are not able to remove most of the traditional and emerging pollutants, the search for a new sustainable and more efficient water treatment method seems to be essential [27].

In the last decade, adsorption processes have gained more and more interest among the possible methods to remove emerging pollutants from water. Compared to conventional wastewater treatment, the adsorption process is characterized by low cost, feasible operation procedure, suitable efficiency and, finally, a large number of potential absorbents [28], among them, materials from natural sources [29,30,31]. The report showed that testings of new absorbents for pharmaceuticals such as antibiotic [32,33], NSAIDs [34,35], and others [36] have increased in the last 5 years.

Among the different potential absorbents, many researchers have employed GO composites and their derivates to absorb different pollutants, such as heavy metals and pharmaceutical compounds [37,38]. Heavy metals like lead (Pb^2+^) and Cadmium (Cd^2+^) have been removed successfully using Alginate-GO Composite Aerogel [38]. Some pharmaceutical compounds like antibiotics [39] (trimethoprim and isoniazid) and antidiabetics (metformin) have also been removed using GO at different pH [39].

Chitosan (CS) has played an essential role in absorbing and eliminating pharmaceutical compounds like antibiotics (amoxicillin, tetracycline, chloramphenicol), non-steroidal anti-inflammatory (diclofenac, ibuprofen, naproxen), and others (pramipexole, carbamazepine, Hydroquinone) [40].

Although the aforementioned materials have a high absorption capacity and are therefore potential means of removing some types of emerging pollutants, it is not certain that they are suitable to remove other types of pollutants. In this sense, the universal absorbent material for emerging pollutants does not seem to exist. For this reason, it is necessary to evaluate the affinity of absorbents to specific materials in order to increase the probability of success in the experiment. To achieve this goal, several authors in the last decade have used in silico calculations as a fundamental tool for the prediction and selection of suitable materials for the absorption of specific new pollutants [41,42,43,44,45,46].

In line with the above described, in this work, the absorption capability of graphene oxide and chitosan toward two specifics emergent pollutants were studied by using the density functional theory (DFT) [47], which is broadly used to understand and predict the adsorption of a specific molecule over a polymeric structure [48,49,50,51,52,53,54,55], proving suitable correlations with the experimental results.

Both amoxicillin (AMOX) and ibuprofen (IBU) were selected due to their occurrence reported in several studies around the world [56,57,58,59,60,61,62], mostly in South America [63,64,65] where the lack of environmental regulations [66] limits the study of occurrence and fate for emerging pollutants. Graphene and chitosan matrices were selected according to the bibliographic reports that reveal good absorption toward compounds with similar structures [67,68].

Relative absorption capability was studied through interaction energy [69] and Gibbs energy concepts, while the nature of molecular interactions was studied by using second-order perturbation theory [70] and energy decomposition analysis (EDA) [70], respectively.

The results obtained herein suggest that in fact, GO and CS could be used as amoxicillin and Ibuprofen absorbents. Likewise, it seems the methodology described herein could be used as a predictive model for the reasonable selection of absorbent materials for a specific pharmaceutical pollutant. Also, according to interaction energy results, CS might enhance the absorption properties of GO toward these kinds of compounds, in line with work reported before [68].

## 2. Materials and Methods

### 2.1. Molecular Models for Pharmaceuticals and Absorbent Structures

The 2D structure of amoxicillin and ibuprofen was retrieved from the PubChem website (https://pubchem.ncbi.nlm.nih.gov, accessed on 5 June 2020). Due to the many dihedral angles in the structures of the pharmaceuticals, the advanced systematic rotor search implemented in the Avogadro software [71] was used to find the lowest energy conformer in each case. Once global minimal structures were found, they were saved as .gjf format and opened with Gaussian view 6 [72], followed by optimization with DFT, specifically the exchange-correlation functional ωB97X-D with basis set 6-311G(2d,p), implemented in G16 for Linux [73]. The dimers of graphene oxide and chitosan were optimized using the same functional and basis set combinations. The minimum energy structures were confirmed by performing frequency calculations. In all cases, no imaginary frequencies were found.

Absorbing materials, i.e., graphene oxide (pyrene, GO-1 and coronene-based, GO-2) and chitosan were simulated as dimers due to the high computational cost of modelling polymeric structures. Nevertheless, several works have represented these types of compounds in these forms [41,74,75,76,77,78].

### 2.2. Pharmaceutical-Dimers Complexes

The minimal structures of pharmaceuticals and dimers were assembled using a clipboard tool implemented in Gaussian view. These images were saved as .gjf and opened with Avogadro Software. Again, a systematic rotor search was used to find the minimum energy structures. The 10 minimum-energy complexes were again saved as .sdf and opened with the Gaussian 16 software; these structures were then optimized at ωB97X-D with the basis set 6-311G(2d,p) and their minimum structures were verified using frequency calculations.

The density functional theory combined with the basis set 6-311G(2d,p) was chosen both for its good time duration and for its agreement with experimental results [79,80]. Moreover, the functional ωB97X-D reproduces very well both the experimental thermodynamic and kinetic parameters in bimolecular reactions [81,82,83,84].

After optimizing the complexes and ensuring that this represented a minimum energy structure, the total binding energies [85] were computed employing the following equation:

(1) $${\Delta E}_{T}=E^{\left(A,B\right)}-\left(E^{A}+E^{B}\right)+\mathrm{BSSE}$$

where EA and EB represent the electronic energy of molecules A and B, respectively; E^(A, B)^ represents the electronic energy of the A,B complex, and BSSE represents the basis set superposition error [85]. A negative value for ${\Delta E}_{T}$ means a favorable interaction between two molecules and an exothermic process. Similarly, the thermodynamics of complexations were studied using the Gibbs equation as follows:

(2) $${\Delta G}_{\mathrm{binding}\;}={\;\Delta H}_{\mathrm{binding}}-{T\Delta S}_{\mathrm{binding}}$$

(3) $${\;\Delta H}_{\mathrm{binding}\;}={\;H}_{\mathrm{complex}}-\left(H_{\mathrm{pharm}}+H_{\mathrm{dimer}}\right)$$

(4) $$\;\;\;\;\;{\;\Delta S}_{\mathrm{binding}\;}={\;S}_{\mathrm{complex}}-\left(S_{\mathrm{pharm}}+S_{\mathrm{dimer}}\right)$$

where H and S are the enthalpy and entropy of complex, pharmaceutical and dimer, respectively, calculated at 298.15 K and 1 atm, retrieved from frequency calculations output.

### 2.3. Electronic Transference, Conceptual Density Functional Theory and Molecular Interactions-Type (NBO Second-Order Perturbation Theory)

In an intermolecular process, the electron flux represents a fundamental parameter to understand the molecular interaction type [85]. The electron flux for each complex was calculated by using Equation (5).

(5) $$\Delta N=\frac{\left({µ}_{B}-{µ}_{A}\right)}{\left(\eta_{A}-\eta_{B}\right)}$$

In Equation (5), η represents the global hardness of both pharmaceuticals and absorbents, respectively, and µ represents the potential electronic of pharmaceutical and absorbents, respectively. A negative value of ΔN means spontaneous electron flows from molecule A to B.

Electronic potentials and global hardness were calculated using the values of both ionization potential (IP) and electronic affinity energy (AE), respectively, according to Equations (6) and (7), already described before [85].

(6) $$\;µ=\frac{-\left(I_{P}+A_{E}\right)}{2}$$

(7) $$\eta \;=\left(\frac{I_{P-A_{E}}}{2}\right)$$

Natural Bond Orbital (NBO) calculation was carried out to estimate the evolution of charge distribution between pharmaceuticals and dimers and complexes. In addition, the binding energies between two atoms were evaluated through the second-order perturbation theory [85], employing Equation (8) using the parameter in the Fock matrix.

(8) $$E_{\mathrm{ij}}{}^{\left(2\right)}=-q_{i}\frac{{\left(F_{\mathrm{ij}}\right)}^{2}}{\left(E_{j-}E_{i}\right)}$$

In this last equation, q_i_ is the donor electronic occupied orbital; E_j_ and E_i_ are orbital energies; and $F_{\mathrm{ij}}$ is out-diagonal Fock matrix element. The greater the value of E_ij_, the greater the interaction between two molecular fragments.

### 2.4. Energy Decomposition Analysis

To estimate the major contribution to the interaction energy, the energy decomposition analysis (EDA) procedure was computed for all complexes studied herein. For this goal, the simple EDA described in Multiwfn software [86] (Tian Lu, Beijing, China) was applied.

In the framework of the Kohn-Sham molecular orbital model [87], the total binding energy (ΔE_T_) can be expressed as the sum of the total interaction energy of the complex (ΔE_int_) and the energy required to prepare the monomers in the arrangement of interaction (ΔE_prep_):

ΔE_T_ = ΔE_int_ + ΔE_prep_ (9)

The total interaction energy was estimated using the following equation:

(10) $${\Delta E}_{\mathrm{int}}{=E}^{\mathrm{Complex}}-\underset{i}{\sum }E_{i}^{\mathrm{Fragments}}$$

The isolated monomers with their conformation of interaction during the complex formation are called fragments.

In turn, the interaction energy can be decomposed into electrostatic (ΔE_elect_), orbital (ΔE_oi_), and Pauli-repulsive (ΔE_Pauli_) energies, followed by the correction term for the error due to the superposition of the basis set used in the calculations.

ΔE_int_ = ΔE_elect_ + ΔE_oi_ + ΔE_Pauli_ + BSSE (11)

The term ΔE_elect_ was calculated as the sum of the energies: nuclear-electron attraction (ΔE_V_), classical inter electron Coulomb repulsion (ΔE_J_), and nuclear-nuclear repulsion (ΔE_Nuc_). This sum equals the energy without exchange-correlation (ΔE_NKVJ_) minus the kinetic energy (ΔE_K_):

ΔE_elect_ = ΔE_V_ + ΔE_J_ + ΔE_Nuc_ = ΔE_NKVJ_ − ΔE_K_ (12)

The orbital interaction energy (ΔE_oi_) was determined by a single point of the complex from the combined initial guess of the wave functions of the fragments. Hence, the orbital interaction energy is the difference of the first iteration energy SCF with the last iteration energy SCF:

ΔE_oi_ = E_SCF,last_ − E_SCF,1st_ (13)

The combination of the wave functions of the fragments was obtained using Multiwfn software [86].

Finally, the Pauli-repulsive energy, ΔE_Pauli_, was estimated using the following equation:

ΔE_Pauli_ = ΔE_int_ − ΔE_e_ − ΔE_oi_ − BSSE (14)

All energies described so far have been determined using the level of theory ωB97X-D/6-311g(2d,p) [73], implemented in the Gaussian 16 package.

On the other hand, the “classical mechanic” interaction energy (ΔU_int_) was estimated employing the potential of pairwise non-covalent interactions between the atoms that constitute the fragments in the complex (previously obtained by DFT):

(15) $${\Delta U}_{\mathrm{int}}=\frac{1}{{4\pi \varepsilon }_{0}}\underset{a\;\epsilon \;A}{\sum }\underset{\;b\;\epsilon \;B}{\sum }\frac{q_{a}q_{b}}{r_{\mathrm{ab}}}+\underset{a\;\epsilon \;A\;}{\sum }\underset{b\;\epsilon \;B}{\sum }{4\varepsilon }_{\mathrm{ab}}\left[{\left(\frac{\sigma_{\mathrm{ab}}}{r_{\mathrm{ab}}}\right)}^{12}-{\left(\frac{\sigma_{\mathrm{ab}}}{r_{\mathrm{ab}}}\right)}^{6}\right]\;$$

where a and b are sites belonging to fragments A and B, respectively, separated by a distance $r_{\mathrm{ab}}$, $q_{a}$ and $q_{b}$ are the partial charges centered on the individual atoms, and $\varepsilon_{0}$ is the electrical permittivity of space. The parameters $\sigma_{\mathrm{ab}}$ and $\varepsilon_{\mathrm{ab}}$ [88], defined as $\sigma_{\mathrm{ab}}{=\sigma }_{a}{+\sigma }_{b}\;$and $\varepsilon_{\mathrm{ab}}=\sqrt{\varepsilon_{a}{+\varepsilon }_{b}\;}$, were obtained using AMBER99 and GAFF force fields.

The first term in Equation (7) is the Coulomb term and accounts for the electrostatic interactions between fragments A and B:

(16) $${\Delta U}_{\mathrm{elect}}=\frac{1}{{4\pi \varepsilon }_{0}}\underset{a\;\epsilon \;A}{\sum }\underset{\;b\;\epsilon \;B}{\sum }\frac{q_{a}q_{b}}{r_{\mathrm{ab}}}$$

$U_{\mathrm{elect}}$ was estimated from the NPA charges [89].

The second term is the Lennard-Jones potential and accounts for the van der Waals interactions (${\Delta U}_{\mathrm{vdW}}$):

(17) $${\Delta U}_{\mathrm{vdW}}=\underset{a\;\epsilon \;A\;}{\sum }\underset{b\;\epsilon \;B}{\sum }{4\varepsilon }_{\mathrm{ab}}\left[{\left(\frac{\sigma_{\mathrm{ab}}}{r_{\mathrm{ab}}}\right)}^{12}-{\left(\frac{\sigma_{\mathrm{ab}}}{r_{\mathrm{ab}}}\right)}^{6}\right]$$

For the resolution of Equations (8) and (9), Multiwfn software (Tian Lu, Beijing, China) was used [86].

## 3. Results

### 3.1. Minimum Molecular Structures

**Figure 1:**
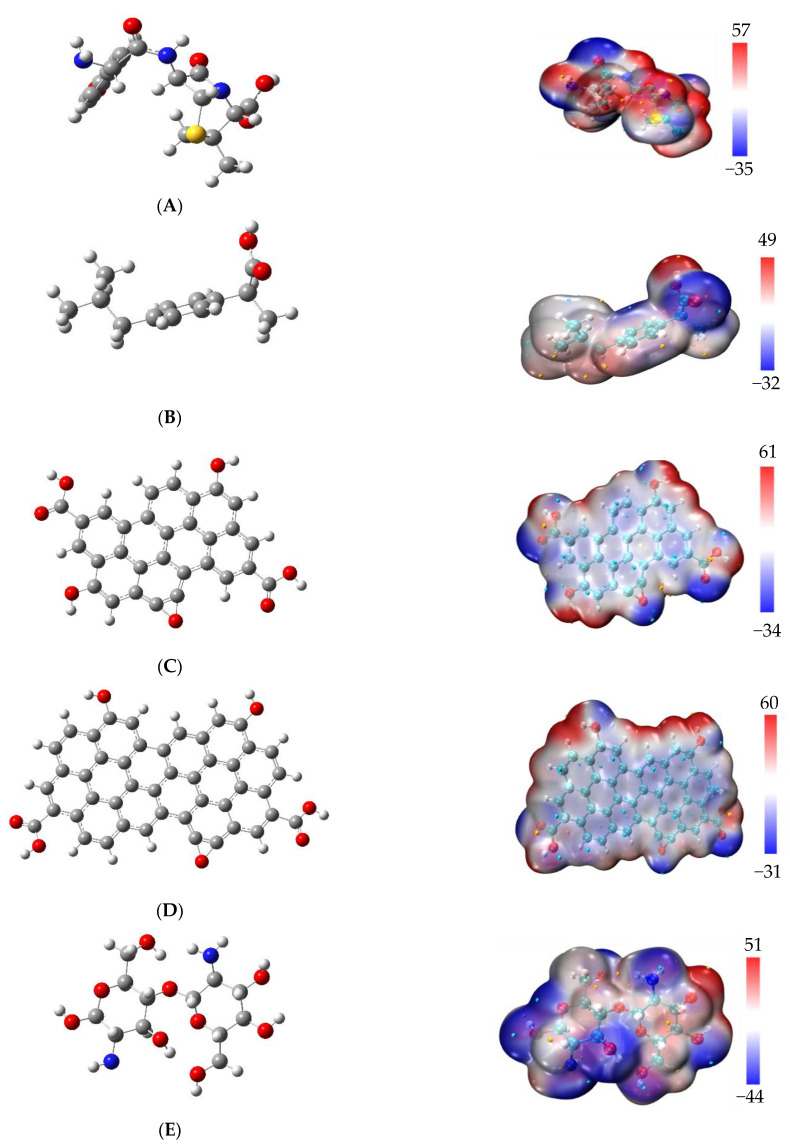
Left: minimum energy structures at ωB97X-D/6-311G(2d,p) level of theory. (**A**) amoxicillin; (**B**) ibuprofen; (**C**) graphene oxide (pyrene, GO-1); (**D**) graphene oxide (coronene, GO-2); and (**E**) chitosan. On the right, electrostatic potential (ESP) surface; scale in kcal/mol.

The minimum-energy structures for pharmaceuticals were selected from the results of the systematic rotor search; the global minimum-energy structures were optimized at ωB97X-D/6-311G(2d,p) and verified using frequency calculations. The global minimum structures for pharmaceuticals along with GO-1, GO-2, and CS are shown in Figure 1 along with their electrostatic potential (ESP) surface.

Figure 1 shows different colors around the molecular structures. The white color indicates the medium electronic density, the blue color indicates the high electron density, and the red color indicates the low electronic density region. According to Figure 1, the maximum values of electronic density follow the decreasing pattern: GO-1 (pyrene) > GO-2 (coronene) > AMOX >CS > IBU, while the order of small electrostatic potentials was CS > AMOX > GO-1 (pyrene) > IBU > GO-2 (coronene). In general, the maximum values (in blue colour) can be associated with O and N atoms, while the minimum values (red color) are associated with hydrogen atoms linked to O or N atoms. In this sense, the results suggest that some van der Waals interactions (including Hy-drug bonds) could be favorable. Nevertheless, the presence of benzene molecules in both pharmaceuticals and Gos could favor the π–π interactions.

Interestingly, in the pharmaceutical compounds studied here, the maximum and minimum electron density for amoxicillin (7.86 Debye) are distributed around the molecule, while for ibuprofen (1.66 Debye) the maximum and minimum electron density are mainly localized at the carboxylic acid moiety. The first look at Figure 1 suggests that the best adsorbent for amoxicillin is CS, whose ESP is close in structure to amoxicillin. In contrast, graphene oxides have a similar ESP to ibuprofen, therefore, these last compounds would be a suitable absorbent for ibuprofen. However, the combination of high surface area with both polar and non-polar moieties suggests a higher absorption spectrum for the Gos than for the chitosan.

Molecular orbitals play a pivotal role in all processes involved in molecular interactions. According to Fukui et al., a chemical interaction can be understood by the analysis of just two orbitals: the frontier orbitals [90]. In a molecular interaction of both frontier orbitals, the highest-energy occupied molecular orbital (HOMO) and the lowest-energy unoccupied molecular orbital (LUMO) must be close together and have similar symmetry to minimize the Gibbs energy of the global process.

**Figure 2:**
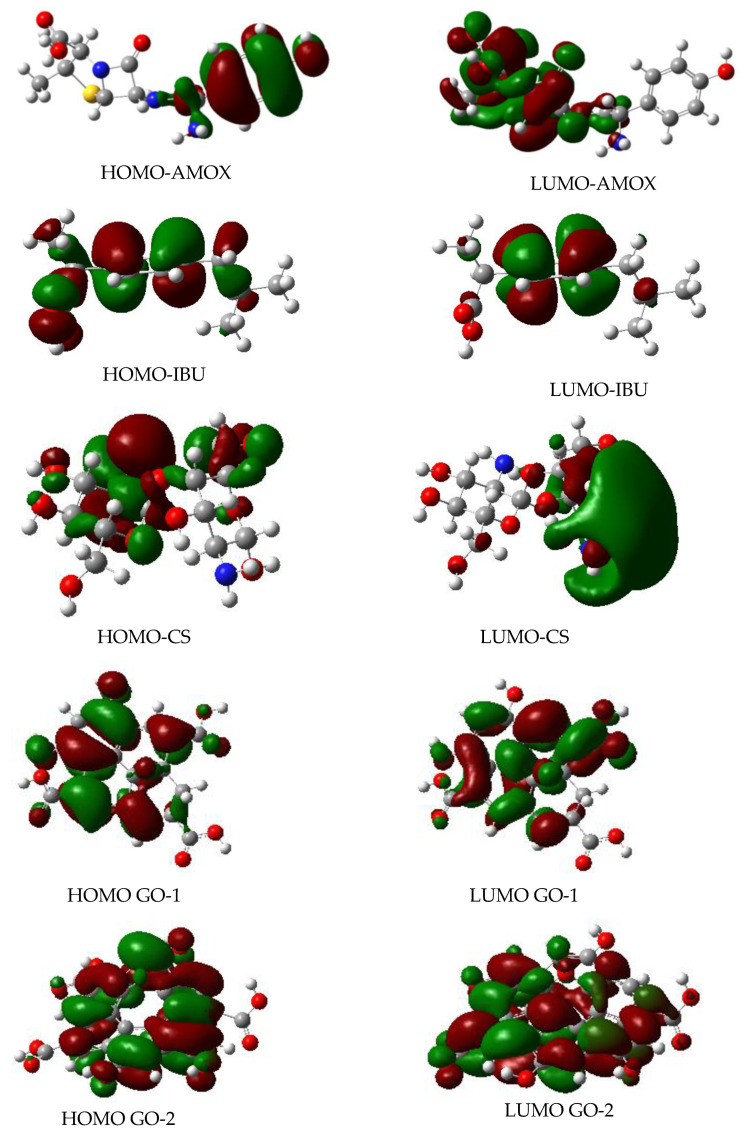
Frontier molecular orbitals for compounds studied in this work. HOMO: Highest occupied molecular orbital; LUMO: lowest unoccupied molecular Orbital obtained at wB97X-D/6311G(2d,p) theory level. AMOX = amoxicillin; IBU = ibuprofen; CS = chitosan; GO-1 = pyrene-based graphene; GO-2 = coronene-based graphene.

Figure 2 shows the frontier molecular orbitals for the entire isolated compounds studied in this work. For the potential absorbents, CS and GOs, a dimer structure was used. The HOMO and LUMO were obtained from the optimized structures at ωB97X-D/6-311G(2d,p) level of theory.

As it can be noted, amoxicillin has the HOMO and LUMO located at different regions in the molecular structure. This behaviour in the frontier orbitals has already been reported before for antimicrobial [91,92], antimicrobial-like [93], and antiprotozoal compounds [94], asserting to be a fundamental molecular characteristic for antimicrobial activity.

In contrast, Ibuprofen and NSAID show frontier orbitals spread around almost the entire molecule, while GOs have the HOMO and LUMO distributed around the entire molecular structure. Interestingly, CS has the HOMO and LUMO located in different molecular regions, similarly to antibiotics and antimicrobials compounds. However, this frontier orbital shape should not surprise us because several works have reported the antimicrobial activity of chitosan polymers and chitosan nanoparticles [95,96,97].

According to the frontier orbitals shape shown in Figure 2, there could be a suitable molecular recognition between pharmaceuticals with CS and GOs. However, two possible combinations stand up due to their higher similitude in their frontier molecular orbitals: amoxicillin-CS and ibuprofen-CS. On the other hand, the complexity in the frontier orbitals in GOs makes it difficult to estimate whether there is molecular recognition with these pharmaceuticals. Nevertheless, the HOMO and LUMO located at the same region, jointly the high surface area, suggest broad absorption spectra.

### 3.2. Complexes Structures of Amoxicillin and Ibuprofen, with Chitosan and Graphene-Oxides

**Figure 3:**
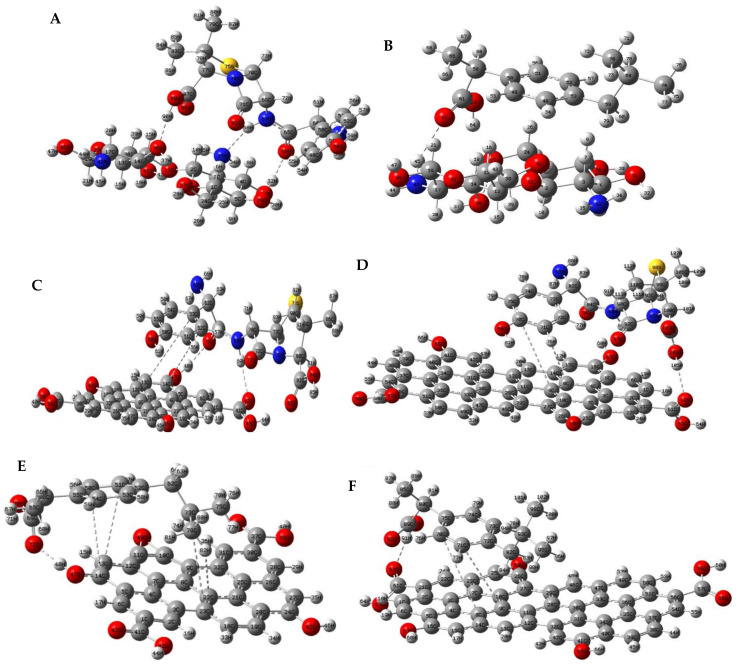
Optimized structures at ωB97X-D/6-311G(2d.p) level of theory for all complexes studied in this work. (**A**) amoxicillin-chitosan; (**B**) ibuprofen-chitosan; (**C**) amoxicillin-GO-1; (**D**) amoxicillin-GO-2; (**E**) ibuprofen-GO-1; (**F**) ibuprofen-GO-2. The dashed line suggests an important molecular interaction.

The minimum-energy complexes for amoxicillin-CS, amoxicillin-GOs, ibuprofen-CS, and ibuprofen-GOs were found by using the systematic rotor search, employing Avogadro Software. The 10 minimum-energy complexes were then optimized using ωB97X-D/6-311G(2d,p) level of theory. However, Figure 3 shows only the global minimum energy structure in each case. For better visualization and understanding, the atomic numbering is shown according to Gaussian software coordinates.

Figure 3A shows the minimum energy structure for the amoxicillin-chitosan complex. The global minimum energy structure suggests a parallel approach between amoxicillin and chitosan-dimer, with mostly van der Waals interactions. The interatomic distance smaller than 3 Å between H(90)—O(36), H(58)-N(33), and O(55)-H(32), is a piece of strong evidence that the van der Waals interactions might be hydrogen bonds in nature. In contrast, the parallel approaching of ibuprofen and chitosan to form the complex ibuprofen-chitosan (Figure 3B), presents just one possible hydrogen bond: O(62)-H(45); the rest of the molecular interactions seem to be dispersion forces in nature.

Figure 3C,D show the minimum energy structures for the interaction between amoxicillin with both GO-1 and GO-2. In both cases, few possible hydrogen bonds are visualized, all of them corresponding to either carboxyl, N-H bond, or carboxylic acid moiety in amoxicillin with the carboxylic acid in the edge of both GO-1 and GO-2. Interestingly, in both cases, the complexes form in parallel approaching with amoxicillin benzene ring to GOs at 2.1 Å; therefore, hydrophobic interactions such as π–π [98,99,100] and σ–π [101,102,103] might be enhanced.

Finally, Figure 3E,F shows the minimum energy complexes for ibuprofen-GOs interaction. In both complexes, the interaction is brought about by the aromatic ring of ibuprofen approaches parallel to graphene oxides structures. In both structures, just one hydrogen bond seems to take place; being the interaction of the carbonyl group of ibuprofen with the carboxylic acid hydrogen into the edge of the GOs. However, the short distances (2.4 Å) between the aromatic rings of ibuprofen and the graphene suggest predominant π–π interactions.

So far, the results suggest an appropriate molecular interaction between amoxicillin-CS, probably driven by van der Waals force, a suitable interaction between amoxicillin-GOs, driven by both VDW and hydrophobic interactions and, finally, interactions between ibuprofen-CS, driven by VDW and hydrophobic molecular interactions.

### 3.3. Energetic and Thermodynamic Parameter for the Complex Formation

An energetic study of the complexes between pharmaceutical (amoxicillin, ibuprofen and tetracycline derivatives) and absorbent (graphene oxides and chitosan) sheds light on the compatibility between pharmaceuticals and absorbents, and the plausibility of the complex. For this reason, concepts such as interaction energy and Gibbs energy were used (see Table 1).

As it is noted in Table 1, all of the complexation enthalpies are negative, indicating exothermic processes. The magnitude of the enthalpy values suggests the presence of dipolar and hydrophobic molecular interactions [104]. Besides, in all cases, the magnitude of the enthalpies indicate an adsorption process is taking place [105,106,107]. Negative values for the complexation ΔS indicate a diminishing in the degree of freedom for molecules involved in the process. Considering the molecular sizes of the absorbent are larger than those of the pharmaceuticals, the small values of entropies are regarded as a consequence of the conversion of translational or rotational degree of freedom in binding movement, mostly for adsorbates (ibuprofen and amoxicillin). Besides, the magnitudes of this parameter are in agreement with values for reported adsorption processes [108,109,110].

Table 1 shows the values of Gibbs energy changes along the absorption process. In all cases, the variation of Gibbs energy is negative, indicating that all complexation processes are spontaneous and exergonic in nature. As it can be noted from Table 1, the complexation of both amoxicillin and ibuprofen are more spontaneous with chitosan than graphene oxides, suggesting chitosan is more appropriate to remove these kinds of pollutant from water. Another important fact is that there is no selectivity between the type of graphene, which means that ibuprofen and amoxicillin could be absorbed on either pyrene or coronene without differences. Finally, Table 1 reveals the total binding energy between pharmaceuticals and absorbents. The negative value of ΔE_T_ for all complexes confirms that there are attractive interactions between amoxicillin and ibuprofen with chitosan or graphene oxides. Moreover, the magnitude of this parameter is coherent with the complexation occurring by a spontaneous and exergonic physisorption process. According to the thermodynamic results, the absorption order is: AMOX-CS > AMOX-GO (pyrene) > AMOX-GO (coronene) > IBU-CS > IBU-GO (coronene) > IBU-GO (pyrene). Interestingly, our results suggest the absorption for amoxicillin and ibuprofen over graphene oxides, in line with the experimental reports [68], showing good predictability of the computational tools.

In addition, to confirm that the results stated above are not isolated ones, the interactions of tetracycline, oxytetracycline, and doxycycline, with chitosan and graphene oxides were carried out by the same methodology. The corresponding minimum-energies figures are shown in Supplementary Materials, jointly their hessian matrixes (Figure S1). Moreover, considering that the adsorption process of these three antibiotics over GO has been already reported experimentally [111], we verified if the methods employed in this manuscript are capable to reproduce, qualitatively, those results.

According to the Nernst [112] equation, ΔG α Ke, and Ke are related to the maximum adsorption (equilibrium concentration) of any adsorbate over an absorbent [113]. Thus, even though Ke depends strongly on the applied adsorption model, the adsorption is driven by ΔG; the smaller the value of ΔG, the greater the equilibrium constant. Moreover, a small concentration of the pollutant in the water (<10 ppm) warrants the fit with any adsorption model.

In this sense, according to ΔG values, the predicted order of adsorption for the three tetracycline-type antibiotics for GO is doxycycline > tetracycline ≅ oxytetracycline. Interestingly, this adsorption order is in agreement with the experimental reports of Gao et al. [111], suggesting this methodology allows us to predict the suitable absorbent for a specific pharmaceutical pollutant.

In line with the last paragraph, the selectivity of any absorbent toward a specific pollutant can be estimated by comparing the ΔG quotient as follows:

(18) $$\frac{{\Delta G}_{A}\;}{{\Delta G}_{B}\;}=\mathrm{Ln}\left(\frac{K_{e1}}{K_{e2}}\right)$$

where A and B are the compared pollutants. For example, the predicted selectivity of CS for AMOX instead of tetracycline is about 66/1. On the other hand, there would be 66 molecules of amoxicillin for each molecule of tetracycline adsorbed in chitosan-based polymers; about 10 molecules of amoxicillin for each molecule of ibuprofen adsorbed in chitosan-based polymers; and in the case of graphene oxides materials, there would be about 24 molecules of amoxicillin for each molecule of ibuprofen, and so on. In contrast, our results predict no selectivity for chitosan toward tetracycline or doxycycline and no selectivity of GO toward tetracycline or oxytetracycline.

### 3.4. Electronic Transference, Conceptual Density Functional Theory and Molecular Interactions-Type (NBO Second-Order Perturbation Theory)

The dual DFT descriptor ΔN was calculated to understand the nature of molecular interaction between the pharmaceuticals and the absorbent models (chitosan or graphene oxides). For this goal, chitosan and graphene-oxides were considered as species B while ibuprofen and amoxicillin compound as species A. Table 2 reveals that, in all cases, the sign of ΔN is negative, suggesting the electron fluxes go from pharmaceuticals toward absorbent, where pharmaceuticals (amoxicillin and ibuprofen) are the donator species while chitosan and graphene oxides are the acceptor species. Likewise, the electronic potential for all complexes is negative, confirming that if they form, they would be energetically stable. These stabilities are supported by the magnitude of global hardness, suggesting the chitosan and graphene oxides appear to be suitable materials for absorbing these types of pharmaceuticals.

Table 3 shows the most important interactions, from the second-order perturbation theory, between pharmaceuticals and each absorbent. The cut-off energy was > 0.20 kcal/mol.

Table 3 reveals that the energetic interactions between amoxicillin and chitosan are mostly dipolar in nature. Some interactions stand up, due to their energy values: LPN33→BD* N67-H68, LPO33→BD* O89-H90, LPO66→BD* O31-H32 and LPO66→BD* O31-H32. The involved atoms and the energy magnitude confirm these interactions are hydrogen bonds in essence. Besides, the lone pair (LP) in atoms of N and O in the chitosan are donators and contribute strongly to the stabilization of the complex through a hydrogen bond. In contrast, the dispersion interactions such as BD C8-C9→ BD* C70-C71, and BD C15-C16→ LP*(1)C84 are less important.

In the same line, the ibuprofen-chitosan is mostly stabilized by dipolar interactions such as LP O62→ BD* N43H45, LP O62→ BD* N43-H45, and a strong hydrogen bond LP O27→ BD* O63-H64; the latter with the highest energy (23.05 kcal/mol). However, some other Van der Waals interaction such as LP O41→ BD* C48-H 54, BD C61-O62→ BD* C14-H18 contribute, in a lesser way, to the stabilization. Comparing the results obtained for amoxicillin, it seems that even though the complexes are mostly stabilized by Van der Waals interactions, the interaction energies in amoxicillin are highest. Therefore, these results support the idea that chitosan is a better absorbent for amoxicillin than ibuprofen.

Table 3 shows the interaction of amoxicillin with GOs. Interestingly, even though the GOs are close in molecular structure, the molecular interactions of amoxicillin with both GO-1 and GO-2 are different in energy. For example, the hydrogen bonds interactions in GO-1 such as LP(1)O42→ BD* N68-H69, LP(1)O76→ BD* O47-H48, and LP(1)O83→ BD* O43-H44 are stronger than the hydrogen bond in GO-2. However, the hydrophobic interactions, mostly π–π, are stronger in GO-2 than in GO-1. Considering the dipolar interactions are, in general, stronger than hydrophobic ones, the result suggests better interaction of amoxicillin with GO-1 than with GO-2.

Finally, Table 3 reveals the interactions of ibuprofen with GO-1 and GO-2, respectively. It can be noted that while in the complex ibuprofen-GO-1(pyrene) there are three classical hydrogen bonds, (LP(1)O72→ O47-H48; BD C69-O70→ BD* O47-H48; and BD C69-O72→ BD* O47-H48), in the complex ibuprofen-GO-2, there is just one hydrogen bond smaller in energy (LP(1)O62→ BD *O92-H91). However, the rest of the interactions are hydrophobic in nature in both complexes.

### 3.5. Energy Decomposition Analysis (EDA)

To gain more insight into the complexation process, an energy decomposition analysis was carried out. This method represents a great tool to understand the nature of molecular bonding. Table 4 shows total binding energy (ΔE_T_) split into electrostatic energy change (ΔE_elect_), orbital interaction energy (ΔE_oi_), Paulli energy (ΔE_Pauli_), interaction energy (ΔE_int_), and preparation energy (ΔE_prep_). In addition, a second EDA, based in classical mechanic, are shown in Supplementary Materials (Table S1).

Table 4 reveals the electrostatic energy is the principal contributor to the stabilization energy. It can be noted that the largest electrostatic energies correspond to the complexes of CS, reinforcing the importance of the hydrogen bond in these interactions. AMOX-CS, having more hydrogen bonds, corresponds to the highest electrostatic interaction change. Interestingly, both amoxicillin and ibuprofen-GOs complexes have more electrostatic interaction with pyrene than coronene, confirming the results of NBO second-order perturbation theory. Besides, the Lowest HOMO energy in pyrene favors the electronic transference from pharmaceuticals, consequently, the electrostatic interactions are being promoted. The second most important contribution depends on the formed complex. For example, for AMOX-CS it is the term of Pauli, while for IBU-CS and pharmaceuticals-GOs, the second most important is the orbital energy. In the first complex (AMOX-CS), both are polar structures with several electronic density maxima spread around the molecules, driving to a considerable electronic repulsion. In the case of ibuprofen, the electronic repulsion is smaller due to both the smallest dipolar momentum and electronic density distribution. The orbital interaction energy change is related to the electron pair bonding, charge transference, and polarization. As ΔN suggests, the electron flux goes from pharmaceutical to graphene oxides. Therefore, the larger the electronic transference, the larger the HOMO–LUMO interactions and, consequently, the greater the ΔE_oi_ [114,115,116,117]. Finally, the preparation energy refers to the electronic cloud deformation due to the presence of another molecule. According to Table 4, amoxicillin, which is more polar than ibuprofen, has a larger ΔE_prep_. In the case of AMOX-GOs complexes, the frontier molecular orbitals must rearrange to be better interact with the frontier orbital of GOs. Therefore, this term is larger for amoxicillin complexes.

## 4. Conclusions

According to results in this study, electrostatic potential surfaces and frontier molecular orbitals analysis can be used to infer if the absorbent (chitosan and graphene oxide) and an adsorbate (amoxicillin and ibuprofen) can drive to molecular recognition. Interaction energy and thermodynamic parameters were calculated using the DFT calculation at ωB97X-D/6-311G(2d,p) level of theory. The negative signs obtained for the energy interactions of all complexes indicate a suitable interaction between pharmaceutical and absorbent (CS and GOs), while the negative sign of enthalpy and Gibbs Energy reveals the presence of strong molecular interactions and the spontaneity of the process. These statements are supported by the results obtained by applying the second-order perturbation theory. The interaction energies for the AMOX complexes were larger than IBU, being the most significant value for the complex AMOX-CS −34.70 kcal/mol, followed by −33.21 kcal/mol AMOX-GO (pyrene) and −31.33 Kcal/mol AMOX-GO (coronene). H, all interaction energies for the complexes suggest GOs and CS could be used as materials for the removal of these kinds of compounds (AMOX and IBU). In addition, the adsorptions over GOs and CS for other antibiotics such as tetracycline, oxytetracycline, and doxycycline were studied employing the same methodology. The results obtained herein are in line with those reported experimentally, suggesting this methodology can predict the adsorption of a specific pharmaceutical pollutant over CS or graphene oxides materials. The agreement of the theoretical results with experimental ones allowed us to estimate the selectivity of the absorbents toward a specific pollutant, by employing the Nernst equation. In this sense, CS is selective toward amoxicillin instead of tetracycline-derivatives. The larger interaction of GOs with amoxicillin than ibuprofen is in line with some reported experimental works; therefore, the methodology employed in this work, based on density functional theory, could be used to find a suitable absorbent for a specific emergent pollutant prior to the experimentations.

## Figures and Tables

**Table 1:** Thermodynamic parameters for the complexation of amoxicillin, ibuprofen, tetracycline, oxytetracycline and doxycycline with chitosan and graphene oxides, calculated at 298.15 K and 1 atm, using the ωB97X-D/6-31G++(d,p) theory level.

Complex	ΔH (kcal/mol)	ΔS (cal/mol)	ΔG (kcal/mol)	ΔE_T_ (kcal/mol)
AMOX-CS	−42.53	−61.78	−24.11	−25.36
AMOX-GO-1	−36.64	−59.55	−18.88	−21.63
AMOX-GO-2	−39.65	−79.53	−15.93	−24.71
IBU-CS	−27.84	−57.45	−10.71	−22.85
IBU-GO-1	−21.06	−50.55	−5.99	−22.30
IBU-GO-2	−24.51	−54.68	−8.21	−19.41
Tetracycline-CS	−21.06	−51.31	−5.76	−22.73
Oxytetracycline-CS	−37.63	−65.33	−18.15	−31.78
doxycycline-CS	−22.98	−52.94	−7.20	−23.78
Tetracycline-GO-1	−22.56	−54.83	−6.22	−23.29
Oxytetracycline-GO-1	−20.26	−48.71	−5.74	−22.95
Doxycycline-GO-1	−29.10	−60.42	−11.09	−26.70
Tetracycline-GO-2	−18.56	−50.83	−3.41	−18.09
Oxytetracycline-GO-2	−16.26	−44.71	−2.93	−18.47
Doxycycline-GO-2	−27.10	−56.42	−10.28	−26.03

**Table 2:** Conceptual DFT molecular descriptors * calculated from frontier orbitals, for the complex drugs-absorbent at ωB97X-D/6-31++G(d,p).

Molecular Descriptor	AMOX-CS	IBU-CS	AMOX-GO-1	AMOX-GO-2	IBU-GO-1	IBU-GO-2
HOMO	−188.77	−196.17	−159.85	−155.27	−161.78	−156.46
LUMO	30.21	29.21	−10.09	−22.89	−11.05	−23.65
µ global	−79.28	−83.48	−84.97	−89.08	−86.41	−90.05
η global	109.49	112.69	74.88	66.19	75.36	66.40
ΔN	−413.36	−154.32	−213.62	−212.89	−145.44	−111.66

* HOMO, LUMO, µ global and η global in kcal/mol.

**Table 3:** The second-order perturbation theory for amoxicillin and ibuprofen with chitosan and graphene-oxides dimers, by using Fock matrix from Natural Bond Orbital (NBO) calculation at the ωB97X-D/6-31G++(d,p) theory level.

Donator	Acceptor	$E_{ij}^{2}\;(\mathrm{kcal}/\mathrm{mol})$	Donator	Acceptor	$E_{ij}^{2}\;(\mathrm{kcal}/\mathrm{mol})$
Amoxcicillin-Chitosan					
BD C14-H18	BD* O89-H90	0.29	LP(2)O66	BD* N33-H34	0.29
BD N33-H34	BD* N67-H68	0.56	LP(2)O66	BD* O31-H32	1.51
LP O29	BD* C48-H54	0.21	LP(2)O66	BD* N33-H34	0.29
LP O31	BD* C49-H55	0.86	LP N67	BD* C4-H8	0.23
LP N33	BD* N67-H68	17.25	LP(1)O88	BD* C2-H10	0.40
LP O36	BD* O89-H90	0.43	LP(1)O88	BD* C12-H15	0.69
LP O33	BD* O89-H90	25.72	LP(2)O88	BD* C12-H15	1.28
BD C65-O66	BD* O31-H32	4.54	LP(1)O91	BD* C1-H6	0.88
BD C71-O91	BD* C2-H10	0.34	LP(1)O91	BD* C4-H8	0.21
BD C87-O88	BD* C2-H10	0.63	LP(2)O91	BD* C4-H8	0.77
BD C87-O88	BD* C12-H15	0.23	BD C8-C9	BD* C70-C71	0.25
BD O89-H90	BD* C14-O36	0.49	BD C13-C14	BD* C72-C73	0.21
LP(1)O66	BD* O31-H32	4.77	BD C15-C16	LP*(1)C84	0.62
LP(2)O66	BD* O31-H32	1.51	LP(1)O67	BD* N88-H89	0.47
Ibuprofen-Chitosan					
LP O27	BD* O63-H64	1.50	BD C49 - H55	BD* C13-H16	0.23
LP O27	BD* O63-H 64	23.05	BD C51 - C52	BD* C5-H9	0.20
LP O41	BD* C 48-H 54	0.20	BD C53 - C59	RY* H7	0.55
LP O41	BD* C 48-H 54	0.75	BD C61 - O62	RY* H18	0.26
BD C48-C49	RY* H16	0.27	BD C61 - O62	BD* C14-H18	0.94
BD C48-C53	RY* H7	0.29	BD O63 - H64	BD* C24-O27	0.36
BD C 48-C53	BD* C3-H7	0.24	LP O62	BD* C14-H18	0.35
LP O62	BD* N43-H45	2.20	LP O62	BD* N43-H45	1.14
Amoxicillin-GO-1					
BD C7-C8	BD* C50-C51	0.41	BD C50-C51	LP(1)C3	0.70
BD C41-O42	BD* N68-H69	1.24	BD C50-C51	LP*(1)C4	0.78
BD C41-O42	BD* C72-O77	1.11	BD C64-O76	BD* O47-H48	3.44
BD O47-H48	BD* C64-O76	0.34	LP(1)O76	BD* O47-H48	13.39
BD O47-H48	BD* C64-O76	0.60	LP(1)O83	BD* O43-H44	2.56
LP(1)O42	BD* N68-H69	3.63	LP(2)O83	BD* O43-H44	2.43
LP(2)O42	BD* N68-H69	3.74	BD* C52-C53	BD* C13-C14	0.26
BD* C7-C8	BD* C54-C55	0.67	BD* C54-C55	BD* C13-C14	0.53
BD*C41-O42	BD* N68-H69	0.29	BD* C64-O76	BD* O47-H48	1.60
Amoxicillin-GO-2					
BD C8-C9	BD* C70-C71	0.25	BD* C41-C42	BD* C74-C75	0.55
BD C13-C14	BD* C72-C73	0.21	BD C61-O62	LP*(1)H105	3.46
BD C15-C16	LP*(1)C84	0.62	LP(1)O62	LP*(1)H105	2.53
LP(1)O67	BD* N88-H89	0.47	LP(2)O62	LP*(1)H105	4.90
LP(2)O67	BD* N88-H89	2.32	BD* C61-O62	LP*(1)H105	3.70
BD* C11-C12	BD* C70-C71	1.85	BD* C41-C42	BD* C74-C75	0.55
IBU-GO-1					
LP(1)O72	BD* O47-H48	6.81	BD C69-O70	BD* O47-H48	0.22
LP(2)O72	BD* O47-H48	11.48	BD C69-O72	BD* C13-H15	0.42
BD C22-C23	BD* C79-H82	0.20	BD C69-O72	BD* O47-H48	0.40
IBU-GO-2					
LP(1)O62	BD* O92-H91	8.38	LP (1)O92	LP*(1)C61	0.32
BD C72-C73	BD* C10-C27	0.22	LP (2)O92	LP*(1)C61	1.26
BD C89-O92	BD* C21-C22	0.38	BD* C89-O92	BD* C1-C6	0.26
LP(2)O90	BD* C21-C22	0.31	BD* C89-O92	BD* C21-C22	0.37

* Antibonding; LP: lone-pair; BD: Bonding

**Table 4:** Decomposition analysis of total binding energy (ΔE_T_) for the complexes studies herein.

Complex	ΔE_elect_ (kcal/mol)	ΔE_oi_ (kcal/mol)	ΔE_Pauli_ (kcal/mol)	ΔE_int_ (kcal/mol)	ΔE_T_ (kcal/mol)	ΔE_prep_ (kcal/mol)
AMOX-CS	−64.44	−32.31	52.37	−34.70	−25.36	9.41
AMOX-GO-1	−26.79	−21.06	7.39	−33.21	−21.63	11.62
AMOX-GO-2	−24.59	−21.82	7.89	−31.33	−24.71	6.31
IBU-CS	−30.23	−19.79	17.26	−25.50	−22.85	2.62
IBU-GO-1	−13.29	−11.95	−3.47	−24.41	−22.30	2.10
IBU-GO-2	−22.32	−12.00	7.24	−23.21	−19.41	3.80

## Data Availability

The data presented in this study are available on request from the corresponding author.

## Supplementary Materials

The following are available online at https://www.mdpi.com/article/10.3390/polym13101620/s1, Figure S1: Minimum structure for the complexes of tetracycline derivatives with CS an GO, respectively. Table S1: Energy Decomposition Analysis for the complexes of Amoxicillin and Ibuprofen with CS and GOs.
