# Supplementary material for: Predicting the Adsorption of Amoxicillin and Ibuprofen on Chitosan and Graphene Oxide Materials: A Density Functional Theory Study
Source: Polymers (Basel). 2021 May 17;13(10):1620. doi: 10.3390/polym13101620 (PMC8156938; doi:10.3390/polym13101620)
Supplement: Supplementary file 1 [file polymers-13-01620-s001.zip › polymers-1211219-supplementary.pdf]

Supplementary Materials

Table S1. Energy Decomposition Analysis for the complexes of Amoxicillin and Ibuprofen with CS and GOs , by Classical Mechanic.

COMPLEX	ΔU_{elect}	ΔU_{vdW}	ΔU_{int}
AMOX-GO-1	-110.7	-53.3	-164.0
AMOX-GO-2	-72.0	-61.7	-133.7
AMOX-QS	-153.3	-20.2	-173.5
IBU-GO-1	-54.9	-43.2	-98.1
IBU-GO-2	-36.3	-59.2	-95.5
IBU-QS	-86.0	-30.8	-116.8

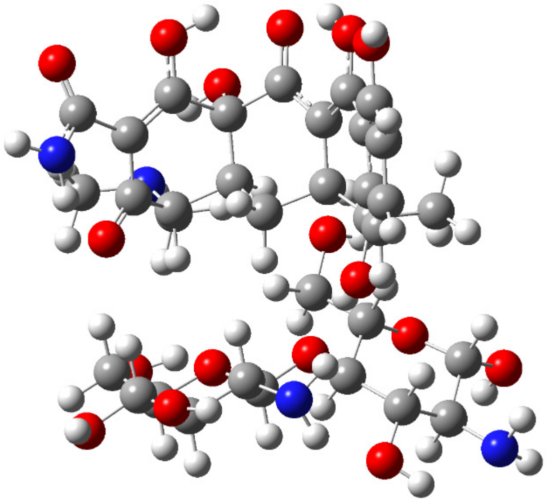

F.S.M.1 CS-Tetracycline

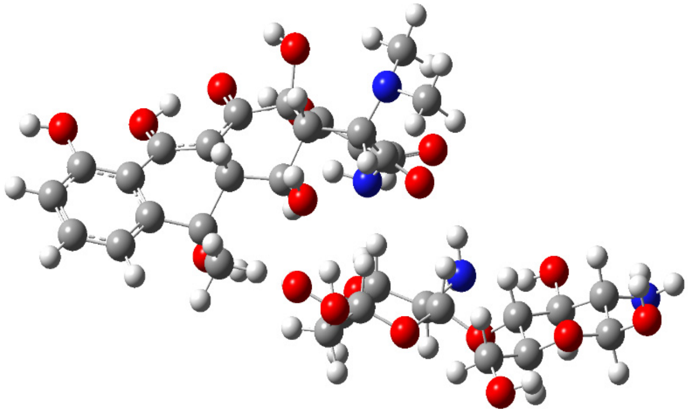

F.S.M.1 CS-Oxitetracycline

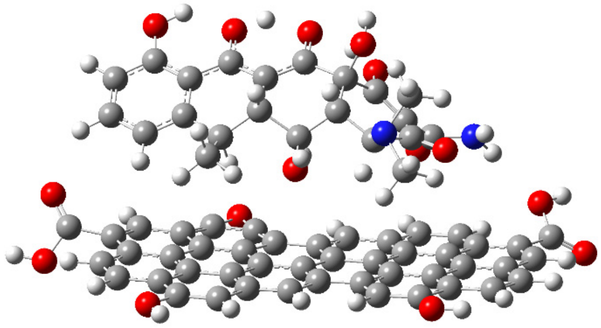

F.S.M.1 GO-Oxitetracycline

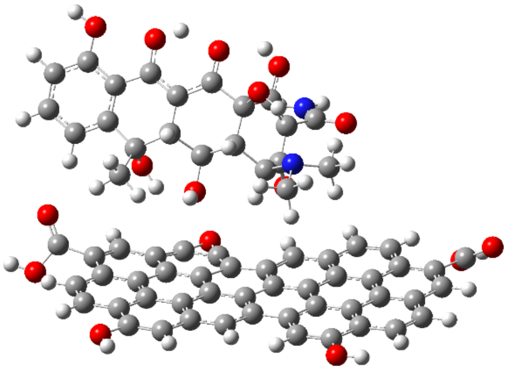

F.S.M.1 GO-doxycycline

Figure S1. Minimum structure for the complexes of tetracycline derivatives with CS an GO, respectively.

2. Hessian Matrix for the new complexes

Gaussian 16: ES64L-G16RevA.03 25-Dec-2016

10-May-2021

****

%nprocshared=32

Will use up to   32 processors via shared memory.

%mem=12GB

----

wb97xd/6-311g(2d,p) geom=connectivity counterpoise=2

1/38=1,57=2,62=2,172=1/1;

2/12=2,17=6,18=5,40=1/2;

1/38=1,53=2,62=2,63=1/22;

3/5=4,6=6,7=102,11=2,25=1,30=1,74=-58/1,2,3;

4//1;

5/5=2,38=5/2;

6/7=2,8=2,9=2,10=2,28=1/1;

1/53=2,62=2,63=1/22;

3/5=7,6=11,7=102,11=2,25=1,30=1,74=-58,82=7/1,2,3;

4//1;

5/5=2,38=5/2;

6/7=2,8=2,9=2,10=2,28=1/1;

1/53=2,62=2,63=1/22(-4);

99/5=1,9=1/99;

----

doxy_GO2_final

Symbolic Z-matrix:

Charge = 0 Multiplicity = 1 in supermolecule

Charge = 0 Multiplicity = 1 in fragment 1.

Charge = 0 Multiplicity = 1 in fragment 2.

C(Fragment=1)	-6.4406	3.16162	0.46839
C(Fragment=1)	-5.6328	2.17876	1.07326
C(Fragment=1)	-4.21851	2.25483	0.95305
C(Fragment=1)	-3.63579	3.30536	0.18633
C(Fragment=1)	-4.47972	4.26788	-0.42438
C(Fragment=1)	-5.87465	4.19265	-0.26694
C(Fragment=1)	-3.3468	1.29091	1.59464
C(Fragment=1)	-2.20093	3.38226	0.02538
C(Fragment=1)	-1.35367	2.43204	0.64336
C(Fragment=1)	-1.94608	1.37918	1.45398
C(Fragment=1)	0.05796	2.51398	0.46159
C(Fragment=1)	0.60268	3.54226	-0.35201
C(Fragment=1)	-0.25177	4.48601	-0.94706
C(Fragment=1)	-1.63497	4.42871	-0.77062
C(Fragment=1)	-2.50154	5.4089	-1.3846
C(Fragment=1)	-3.84809	5.32039	-1.20716
H(Fragment=1)	-2.04563	6.19841	-1.9742
H(Fragment=1)	-7.52597	3.12775	0.59508
H(Fragment=1)	-6.50405	4.9572	-0.73356
H(Fragment=1)	0.17496	5.28327	-1.56129
C(Fragment=1)	-6.22334	1.06394	1.81107
C(Fragment=1)	-5.45222	0.14591	2.44843
C(Fragment=1)	-4.01699	0.23554	2.38072
H(Fragment=1)	-5.88013	-0.69195	3.00976
C(Fragment=1)	-3.09909	-0.57652	2.9453
C(Fragment=1)	-1.71643	-0.47506	2.80368
C(Fragment=1)	-1.01825	0.4294	2.09362

C(Fragment=1)	0.94837	1.55762	1.09451
C(Fragment=1)	0.42252	0.51879	1.9261
C(Fragment=1)	2.33955	1.63814	0.90061
C(Fragment=1)	2.89447	2.67183	0.04998
C(Fragment=1)	2.05002	3.61202	-0.56846
C(Fragment=1)	3.20934	0.701	1.54176
C(Fragment=1)	4.31113	2.72967	-0.15947
C(Fragment=1)	5.18433	1.76769	0.4765
C(Fragment=1)	4.63729	0.75904	1.33372
C(Fragment=1)	6.5755	1.82603	0.2543
C(Fragment=1)	7.11826	2.85355	-0.59835
C(Fragment=1)	6.29835	3.76396	-1.19307
C(Fragment=1)	4.87587	3.72134	-0.98804
C(Fragment=1)	3.98233	4.65868	-1.59771
C(Fragment=1)	2.61488	4.6123	-1.40051
H(Fragment=1)	1.97834	5.35332	-1.88896
H(Fragment=1)	8.19768	2.88015	-0.751
H(Fragment=1)	6.69979	4.54674	-1.83895
C(Fragment=1)	1.26629	-0.38415	2.55511
C(Fragment=1)	2.66953	-0.30151	2.38062
H(Fragment=1)	0.85951	-1.17633	3.19039
C(Fragment=1)	3.56009	-1.227	3.03661
C(Fragment=1)	4.90625	-1.16061	2.84404
C(Fragment=1)	5.48506	-0.18148	1.96112
H(Fragment=1)	3.12263	-2.01067	3.66501
H(Fragment=1)	5.56845	-1.87417	3.34321
C(Fragment=1)	7.41903	0.86141	0.87567
H(Fragment=1)	8.49818	0.90873	0.69646
C(Fragment=1)	6.89094	-0.11978	1.6911

C(Fragment=1)	7.8574	-1.08563	2.26722
O(Fragment=1)	8.97461	-0.88541	2.68311
O(Fragment=1)	7.37499	-2.38146	2.26018
H(Fragment=1)	8.03891	-3.02889	2.62874
C(Fragment=1)	-7.69497	0.86462	1.83005
O(Fragment=1)	-8.32008	-0.11379	1.48494
O(Fragment=1)	-8.36159	1.95809	2.32159
H(Fragment=1)	-9.3494	1.81371	2.36504
O(Fragment=1)	4.42635	5.66239	-2.42191
H(Fragment=1)	5.41646	5.65492	-2.51779
O(Fragment=1)	-4.74597	6.20442	-1.7496
H(Fragment=1)	-4.29739	6.91896	-2.27222
O(Fragment=1)	-2.29776	-1.54792	3.6008
O(Fragment=2)	-0.54091	0.00449	-1.47915
O(Fragment=2)	1.19701	-4.06754	-3.09992
O(Fragment=2)	-0.60366	-5.46968	-1.47249
O(Fragment=2)	1.62718	-5.14574	-0.16736
O(Fragment=2)	-3.05552	-5.52575	-1.08452
O(Fragment=2)	4.20214	-1.5076	-1.30472
O(Fragment=2)	-5.6261	-5.6211	-0.77232
O(Fragment=2)	3.23635	-3.7248	1.85849
N(Fragment=2)	2.22807	-0.5572	-3.18018
N(Fragment=2)	5.02638	-3.45912	0.47884
C(Fragment=2)	0.61126	-2.03614	-2.09827
C(Fragment=2)	-1.91733	-2.01591	-1.72366
C(Fragment=2)	-0.55356	-1.41009	-1.28768
C(Fragment=2)	0.72141	-3.54924	-1.84921
C(Fragment=2)	1.89822	-1.20449	-1.87036
C(Fragment=2)	-3.08819	-1.30504	-0.992

C(Fragment=2)	-1.86209	-3.49905	-1.47868
C(Fragment=2)	-0.65278	-4.21175	-1.59034
C(Fragment=2)	1.72615	-3.94571	-0.75554
C(Fragment=2)	-4.34715	-2.13436	-0.82523
C(Fragment=2)	3.0682	-1.93828	-1.20682
C(Fragment=2)	-3.04101	-4.19048	-1.17804
C(Fragment=2)	2.76723	-3.14574	-0.41716
C(Fragment=2)	-4.30676	-3.56029	-0.917
C(Fragment=2)	-3.40537	0.01333	-1.71038
C(Fragment=2)	-5.54344	-1.50782	-0.49473
C(Fragment=2)	3.03697	0.683	-2.98016
C(Fragment=2)	2.92798	-1.49647	-4.10721
C(Fragment=2)	-5.51266	-4.27717	-0.69984
C(Fragment=2)	3.65743	-3.46401	0.7408
C(Fragment=2)	-6.72217	-2.24426	-0.26839
C(Fragment=2)	-6.72178	-3.62386	-0.37788
H(Fragment=2)	0.34798	-1.94286	-3.19885
H(Fragment=2)	-2.05114	-1.858	-2.82609
H(Fragment=2)	-0.40128	-1.50657	-0.18662
H(Fragment=2)	1.64761	-0.36622	-1.1395
H(Fragment=2)	-2.74537	-1.06028	0.05405
H(Fragment=2)	-3.73685	-0.14821	-2.74086
H(Fragment=2)	-2.51731	0.66653	-1.73304
H(Fragment=2)	-4.19086	0.5829	-1.19616
H(Fragment=2)	-0.35718	0.2411	-2.42122
H(Fragment=2)	1.22364	-5.0599	-3.08252
H(Fragment=2)	-5.585	-0.41958	-0.40935
H(Fragment=2)	2.45599	1.40629	-2.38507
H(Fragment=2)	4.0046	0.51467	-2.47463

H(Fragment=2) 3.23383 1.14407 -3.95995

H(Fragment=2) 3.97629 -1.70277 -3.83716

H(Fragment=2) 2.39157 -2.46177 -4.1494

H(Fragment=2) 2.90914 -1.06133 -5.1184

H(Fragment=2) 0.83556 -5.7188 -0.47784

H(Fragment=2) -2.08001 -5.98232 -1.23931

H(Fragment=2) -7.64156 -1.71045 0.00955

H(Fragment=2) -7.61933 -4.21919 -0.21465

H(Fragment=2) 5.38321 -3.00753 -0.35935

H(Fragment=2) 5.66825 -3.45334 1.26558

H(Fragment=2) -4.76635 -6.10831 -0.97637

Stoichiometry C68H40N2O15

Framework group C1[X(C68H40N2O15)]

Deg. of freedom 369

Full point group C1 NOp 1

Largest Abelian subgroup C1 NOp 1

Largest concise Abelian subgroup C1 NOp 1

Standard orientation:

Center Number	Atomic Number	Atomic Type	Coordinates (Angstroms) X	Y	Z
1	6	0	-6.440598	3.161625	0.468385
2	6	0	-5.632801	2.178755	1.073262
3	6	0	-4.218513	2.254833	0.953046
4	6	0	-3.635794	3.305362	0.186326
5	6	0	-4.479720	4.267877	-0.424376
6	6	0	-5.874647	4.192653	-0.266936

7	6	0	-3.346797	1.290908	1.594644
8	6	0	-2.200929	3.382264	0.025375
9	6	0	-1.353666	2.432037	0.643356
10	6	0	-1.946080	1.379178	1.453979
11	6	0	0.057959	2.513976	0.461589
12	6	0	0.602679	3.542257	-0.352006
13	6	0	-0.251765	4.486011	-0.947062
14	6	0	-1.634966	4.428712	-0.770619
15	6	0	-2.501544	5.408902	-1.384597
16	6	0	-3.848089	5.320394	-1.207155
17	1	0	-2.045628	6.198413	-1.974195
18	1	0	-7.525974	3.127753	0.595081
19	1	0	-6.504052	4.957202	-0.733557
20	1	0	0.174957	5.283272	-1.561291
21	6	0	-6.223344	1.063938	1.811065
22	6	0	-5.452217	0.145910	2.448433
23	6	0	-4.016985	0.235537	2.380716
24	1	0	-5.880132	-0.691945	3.009765
25	6	0	-3.099087	-0.576522	2.945298
26	6	0	-1.716433	-0.475056	2.803682
27	6	0	-1.018251	0.429402	2.093620
28	6	0	0.948371	1.557622	1.094509
29	6	0	0.422523	0.518786	1.926101
30	6	0	2.339545	1.638144	0.900612
31	6	0	2.894465	2.671825	0.049979
32	6	0	2.050019	3.612024	-0.568457
33	6	0	3.209344	0.701004	1.541761
34	6	0	4.311131	2.729665	-0.159473
35	6	0	5.184326	1.767691	0.476501

36	6	0	4.637291	0.759043	1.333719
37	6	0	6.575504	1.826032	0.254295
38	6	0	7.118257	2.853554	-0.598348
39	6	0	6.298355	3.763961	-1.193068
40	6	0	4.875872	3.721337	-0.988039
41	6	0	3.982325	4.658680	-1.597707
42	6	0	2.614876	4.612303	-1.400509
43	1	0	1.978336	5.353315	-1.888959
44	1	0	8.197685	2.880151	-0.750998
45	1	0	6.699795	4.546742	-1.838952
46	6	0	1.266290	-0.384148	2.555106
47	6	0	2.669532	-0.301505	2.380615
48	1	0	0.859507	-1.176329	3.190388
49	6	0	3.560089	-1.227003	3.036609
50	6	0	4.906254	-1.160605	2.844042
51	6	0	5.485055	-0.181475	1.961117
52	1	0	3.122625	-2.010673	3.665005
53	1	0	5.568450	-1.874171	3.343207
54	6	0	7.419027	0.861409	0.875669
55	1	0	8.498184	0.908731	0.696463
56	6	0	6.890937	-0.119775	1.691096
57	6	0	7.857399	-1.085628	2.267215
58	8	0	8.974611	-0.885410	2.683106
59	8	0	7.374992	-2.381459	2.260184
60	1	0	8.038906	-3.028891	2.628742
61	6	0	-7.694966	0.864617	1.830048
62	8	0	-8.320080	-0.113791	1.484936
63	8	0	-8.361586	1.958086	2.321588
64	1	0	-9.349398	1.813709	2.365040

65	8	0	4.426352	5.662389	-2.421908
66	1	0	5.416455	5.654922	-2.517791
67	8	0	-4.745974	6.204423	-1.749598
68	1	0	-4.297393	6.918960	-2.272218
69	8	0	-2.297756	-1.547919	3.600795
70	8	0	-0.540911	0.004486	-1.479145
71	8	0	1.197009	-4.067541	-3.099916
72	8	0	-0.603655	-5.469676	-1.472487
73	8	0	1.627175	-5.145744	-0.167356
74	8	0	-3.055516	-5.525749	-1.084523
75	8	0	4.202137	-1.507599	-1.304721
76	8	0	-5.626102	-5.621100	-0.772322
77	8	0	3.236351	-3.724796	1.858485
78	7	0	2.228070	-0.557200	-3.180179
79	7	0	5.026377	-3.459119	0.478837
80	6	0	0.611258	-2.036137	-2.098271
81	6	0	-1.917331	-2.015910	-1.723658
82	6	0	-0.553557	-1.410085	-1.287684
83	6	0	0.721409	-3.549242	-1.849205
84	6	0	1.898221	-1.204491	-1.870362
85	6	0	-3.088193	-1.305043	-0.992003
86	6	0	-1.862090	-3.499045	-1.478682
87	6	0	-0.652778	-4.211753	-1.590343
88	6	0	1.726148	-3.945707	-0.755536
89	6	0	-4.347148	-2.134363	-0.825231
90	6	0	3.068195	-1.938281	-1.206823
91	6	0	-3.041011	-4.190485	-1.178037
92	6	0	2.767230	-3.145743	-0.417155
93	6	0	-4.306762	-3.560290	-0.917003

94	6	0	-3.405369	0.013331	-1.710382
95	6	0	-5.543441	-1.507823	-0.494725
96	6	0	3.036972	0.682996	-2.980158
97	6	0	2.927979	-1.496465	-4.107206
98	6	0	-5.512657	-4.277169	-0.699841
99	6	0	3.657431	-3.464008	0.740797
100	6	0	-6.722166	-2.244255	-0.268387
101	6	0	-6.721784	-3.623857	-0.377876
102	1	0	0.347981	-1.942863	-3.198851
103	1	0	-2.051138	-1.857995	-2.826092
104	1	0	-0.401276	-1.506569	-0.186615
105	1	0	1.647610	-0.366220	-1.139498
106	1	0	-2.745371	-1.060282	0.054047
107	1	0	-3.736846	-0.148211	-2.740856
108	1	0	-2.517312	0.666525	-1.733040
109	1	0	-4.190861	0.582901	-1.196160
110	1	0	-0.357184	0.241096	-2.421220
111	1	0	1.223641	-5.059904	-3.082520
112	1	0	-5.585000	-0.419584	-0.409351
113	1	0	2.455994	1.406294	-2.385071
114	1	0	4.004605	0.514669	-2.474631
115	1	0	3.233826	1.144068	-3.959953
116	1	0	3.976290	-1.702768	-3.837159
117	1	0	2.391571	-2.461766	-4.149404
118	1	0	2.909135	-1.061329	-5.118401
119	1	0	0.835564	-5.718796	-0.477843
120	1	0	-2.080008	-5.982320	-1.239312
121	1	0	-7.641557	-1.710448	0.009554
122	1	0	-7.619328	-4.219187	-0.214653

123	1	0	5.383211	-3.007531	-0.359349
124	1	0	5.668247	-3.453337	1.265582
125	1	0	-4.766352	-6.108308	-0.976369

Gaussian 16: ES64L-G16RevA.03 25-Dec-2016

10-May-2021

****

%nprocshared=32

Will use up to 32 processors via shared memory.

%mem=12GB

----

wb97xd/6-311g(2d,p) counterpoise=2

1/38=1,62=2,172=1/1;

2/12=2,17=6,18=5,40=1/2;

1/38=1,53=2,62=2,63=1/22;

3/5=4,6=6,7=102,11=2,25=1,30=1,74=-58/1,2,3;

4//1;

5/5=2,38=5/2;

6/7=2,8=2,9=2,10=2,28=1/1;

1/53=2,62=2,63=1/22;

3/5=7,6=11,7=102,11=2,25=1,30=1,74=-58,82=7/1,2,3;

4//1;

5/5=2,38=5/2;

6/7=2,8=2,9=2,10=2,28=1/1;

1/53=2,62=2,63=1/22(-4);

99/5=1,9=1/99;

----

oxitetracycline_GO2_counterpoise

Symbolic Z-matrix:

Charge = 0 Multiplicity = 1 in supermolecule

Charge = 0 Multiplicity = 1 in fragment 1.

Charge = 0 Multiplicity = 1 in fragment 2.

C(Fragment=1)	-3.95483	5.37633	0.53402
C(Fragment=1)	-3.46633	4.1895	1.11402
C(Fragment=1)	-2.10689	3.8196	0.92998
C(Fragment=1)	-1.26369	4.63167	0.11877
C(Fragment=1)	-1.78957	5.81505	-0.46028
C(Fragment=1)	-3.12837	6.18108	-0.23731
C(Fragment=1)	-1.5492	2.63917	1.55885
C(Fragment=1)	0.10989	4.24685	-0.11733
C(Fragment=1)	0.63467	3.06339	0.45356
C(Fragment=1)	-0.21454	2.25866	1.31757
C(Fragment=1)	1.97991	2.6789	0.17963
C(Fragment=1)	2.79167	3.50179	-0.64432
C(Fragment=1)	2.26179	4.68688	-1.18359
C(Fragment=1)	0.94238	5.0707	-0.94086
C(Fragment=1)	0.40341	6.28244	-1.51507
C(Fragment=1)	-0.89342	6.62108	-1.27685
H(Fragment=1)	1.05889	6.89523	-2.12637
H(Fragment=1)	-4.98865	5.6827	0.71107
H(Fragment=1)	-3.50687	7.10732	-0.68126
H(Fragment=1)	2.89528	5.32056	-1.80986
C(Fragment=1)	-4.33704	3.31584	1.89808
C(Fragment=1)	-3.85159	2.23656	2.56243
C(Fragment=1)	-2.46029	1.88469	2.44454

H(Fragment=1)	-4.48895	1.5843	3.17107
C(Fragment=1)	-1.80039	0.85873	3.02181
C(Fragment=1)	-0.47702	0.48833	2.77119
C(Fragment=1)	0.39243	1.05493	1.91712
C(Fragment=1)	2.52642	1.44629	0.71808
C(Fragment=1)	1.73272	0.61335	1.56924
C(Fragment=1)	3.83543	1.04457	0.39781
C(Fragment=1)	4.66854	1.88817	-0.4351
C(Fragment=1)	4.17061	3.10378	-0.93892
C(Fragment=1)	4.34696	-0.19754	0.89162
C(Fragment=1)	6.00699	1.47698	-0.74008
C(Fragment=1)	6.51768	0.21954	-0.23984
C(Fragment=1)	5.68917	-0.62345	0.57068
C(Fragment=1)	7.83167	-0.1797	-0.55702
C(Fragment=1)	8.66038	0.67299	-1.37132
C(Fragment=1)	8.18243	1.85927	-1.8394
C(Fragment=1)	6.84367	2.28655	-1.5363
C(Fragment=1)	6.3034	3.52193	-2.01717
C(Fragment=1)	5.01105	3.92332	-1.73508
H(Fragment=1)	4.64828	4.8753	-2.12853
H(Fragment=1)	9.67387	0.34147	-1.59997
H(Fragment=1)	8.80205	2.51278	-2.4561
C(Fragment=1)	2.22157	-0.59586	2.03573
C(Fragment=1)	3.52948	-1.02341	1.69655
H(Fragment=1)	1.59119	-1.25298	2.65296
C(Fragment=1)	4.0408	-2.29159	2.153
C(Fragment=1)	5.3056	-2.68909	1.84502
C(Fragment=1)	6.17862	-1.85875	1.05402
H(Fragment=1)	3.37679	-2.9279	2.7468

H(Fragment=1)	5.6839	-3.65277	2.20427
C(Fragment=1)	8.31454	-1.42621	-0.06668
H(Fragment=1)	9.33172	-1.7395	-0.32816
C(Fragment=1)	7.51976	-2.23799	0.7186
C(Fragment=1)	8.14472	-3.51222	1.15627
O(Fragment=1)	8.96726	-4.17746	0.56682
O(Fragment=1)	7.73543	-3.91331	2.4071
H(Fragment=1)	8.18308	-4.75887	2.69365
C(Fragment=1)	-5.8016	3.55829	1.91847
O(Fragment=1)	-6.68141	2.84711	1.48505
O(Fragment=1)	-6.11978	4.75106	2.5146
H(Fragment=1)	-7.1051	4.90864	2.55448
O(Fragment=1)	7.03337	4.38601	-2.79449
H(Fragment=1)	7.95504	4.0506	-2.96005
O(Fragment=1)	-1.48928	7.74801	-1.78306
H(Fragment=1)	-0.8612	8.29073	-2.32722
O(Fragment=1)	-1.31052	-0.30253	3.6671
O(Fragment=2)	-1.97228	-0.2709	0.16224
O(Fragment=2)	-1.21423	-3.79488	-3.00413
O(Fragment=2)	-4.53367	-0.64301	1.08765
O(Fragment=2)	-3.62225	-4.88705	-2.27822
O(Fragment=2)	-1.58277	-5.96947	-1.16968
O(Fragment=2)	-6.04577	-4.20012	-2.03108
O(Fragment=2)	0.46987	-2.92068	1.73317
O(Fragment=2)	-8.31344	-3.55162	-1.90564
O(Fragment=2)	1.3436	-6.07138	0.84635
N(Fragment=2)	1.09088	-2.43984	-1.54932
N(Fragment=2)	-0.63748	-6.3117	1.93273
C(Fragment=2)	-1.32455	-2.16849	-1.21201

C(Fragment=2)	-3.73717	-1.38629	-1.12579
C(Fragment=2)	-2.41883	-1.54294	-0.32571
C(Fragment=2)	-1.7372	-3.57195	-1.69509
C(Fragment=2)	0.04919	-2.16559	-0.49783
C(Fragment=2)	-4.76509	-0.51888	-0.32497
C(Fragment=2)	-4.20863	-2.75249	-1.52177
C(Fragment=2)	-3.26754	-3.76021	-1.87656
C(Fragment=2)	-1.21763	-4.72974	-0.81948
C(Fragment=2)	-6.2321	-0.93591	-0.4463
C(Fragment=2)	0.07245	-3.21899	0.62875
C(Fragment=2)	-5.57106	-3.04184	-1.57791
C(Fragment=2)	-0.40617	-4.55586	0.25285
C(Fragment=2)	-4.6066	0.95236	-0.73814
C(Fragment=2)	-6.6034	-2.12903	-1.11127
C(Fragment=2)	-7.20915	-0.11805	0.12123
C(Fragment=2)	1.59934	-1.14754	-2.10752
C(Fragment=2)	2.23595	-3.25443	-1.04527
C(Fragment=2)	-7.98561	-2.41636	-1.22804
C(Fragment=2)	0.19406	-5.69497	1.00749
C(Fragment=2)	-8.56923	-0.43897	0.00834
C(Fragment=2)	-8.97318	-1.57874	-0.67927
H(Fragment=2)	-1.22021	-1.53938	-2.14046
H(Fragment=2)	-3.52082	-0.83529	-2.08155
H(Fragment=2)	-2.58081	-2.11335	0.62219
H(Fragment=2)	0.19151	-1.161	-0.00472
H(Fragment=2)	-3.57753	1.3066	-0.60833
H(Fragment=2)	-5.24076	1.60484	-0.113
H(Fragment=2)	-4.90407	1.12446	-1.77784
H(Fragment=2)	-1.80657	0.36575	-0.56543

H(Fragment=2)	-0.22538	-3.57947	-3.00344
H(Fragment=2)	-3.6665	-0.22499	1.33434
H(Fragment=2)	-6.91478	0.78451	0.67488
H(Fragment=2)	0.76396	-0.55868	-2.51336
H(Fragment=2)	2.14904	-0.52121	-1.38522
H(Fragment=2)	2.27946	-1.36885	-2.94707
H(Fragment=2)	2.75046	-2.83797	-0.16516
H(Fragment=2)	1.89457	-4.27846	-0.78965
H(Fragment=2)	2.98012	-3.37316	-1.84886
H(Fragment=2)	-2.21676	-5.98539	-1.99378
H(Fragment=2)	-5.29092	-4.87566	-2.3436
H(Fragment=2)	-9.3154	0.21752	0.46271
H(Fragment=2)	-10.02606	-1.82298	-0.78101
H(Fragment=2)	-1.57621	-6.0042	2.1077
H(Fragment=2)	-0.29454	-7.07606	2.49192
H(Fragment=2)	-9.29654	-3.70158	-1.94168

Stoichiometry C68H40N2O16

Framework group C1[X(C68H40N2O16)]

Deg. of freedom 372

Full point group C1 NOp 1

Largest Abelian subgroup C1 NOp 1

Largest concise Abelian subgroup C1 NOp 1

Standard orientation:

Center Number	Atomic Number	Atomic Type	Coordinates (Angstroms) X	Y	Z
1	6	0	-3.954831	5.376327	0.534021

2	6	0	-3.466334	4.189502	1.114018
3	6	0	-2.106888	3.819595	0.929981
4	6	0	-1.263689	4.631671	0.118767
5	6	0	-1.789572	5.815049	-0.460283
6	6	0	-3.128369	6.181078	-0.237306
7	6	0	-1.549201	2.639167	1.558850
8	6	0	0.109891	4.246852	-0.117327
9	6	0	0.634665	3.063392	0.453559
10	6	0	-0.214535	2.258661	1.317574
11	6	0	1.979906	2.678897	0.179634
12	6	0	2.791668	3.501793	-0.644323
13	6	0	2.261791	4.686881	-1.183592
14	6	0	0.942379	5.070700	-0.940862
15	6	0	0.403411	6.282444	-1.515073
16	6	0	-0.893418	6.621083	-1.276851
17	1	0	1.058886	6.895225	-2.126368
18	1	0	-4.988645	5.682697	0.711072
19	1	0	-3.506872	7.107323	-0.681260
20	1	0	2.895279	5.320557	-1.809861
21	6	0	-4.337038	3.315844	1.898080
22	6	0	-3.851587	2.236558	2.562426
23	6	0	-2.460293	1.884689	2.444544
24	1	0	-4.488951	1.584297	3.171072
25	6	0	-1.800390	0.858728	3.021813
26	6	0	-0.477024	0.488334	2.771193
27	6	0	0.392428	1.054933	1.917121
28	6	0	2.526422	1.446289	0.718077
29	6	0	1.732715	0.613349	1.569240
30	6	0	3.835425	1.044567	0.397811

31	6	0	4.668537	1.888172	-0.435104
32	6	0	4.170609	3.103784	-0.938916
33	6	0	4.346964	-0.197544	0.891623
34	6	0	6.006986	1.476982	-0.740078
35	6	0	6.517680	0.219543	-0.239839
36	6	0	5.689165	-0.623448	0.570683
37	6	0	7.831670	-0.179704	-0.557024
38	6	0	8.660385	0.672991	-1.371321
39	6	0	8.182430	1.859266	-1.839397
40	6	0	6.843670	2.286551	-1.536298
41	6	0	6.303403	3.521928	-2.017174
42	6	0	5.011045	3.923315	-1.735075
43	1	0	4.648284	4.875304	-2.128528
44	1	0	9.673874	0.341465	-1.599974
45	1	0	8.802051	2.512779	-2.456100
46	6	0	2.221573	-0.595858	2.035733
47	6	0	3.529475	-1.023408	1.696548
48	1	0	1.591186	-1.252979	2.652961
49	6	0	4.040797	-2.291588	2.152997
50	6	0	5.305604	-2.689087	1.845017
51	6	0	6.178623	-1.858745	1.054023
52	1	0	3.376794	-2.927904	2.746799
53	1	0	5.683904	-3.652773	2.204274
54	6	0	8.314540	-1.426207	-0.066682
55	1	0	9.331720	-1.739501	-0.328158
56	6	0	7.519764	-2.237994	0.718601
57	6	0	8.144721	-3.512222	1.156268
58	8	0	8.967257	-4.177463	0.566816
59	8	0	7.735434	-3.913313	2.407104

60	1	0	8.183080	-4.758872	2.693648
61	6	0	-5.801597	3.558286	1.918467
62	8	0	-6.681412	2.847111	1.485053
63	8	0	-6.119782	4.751061	2.514596
64	1	0	-7.105098	4.908638	2.554475
65	8	0	7.033370	4.386009	-2.794486
66	1	0	7.955036	4.050601	-2.960047
67	8	0	-1.489277	7.748006	-1.783061
68	1	0	-0.861199	8.290730	-2.327221
69	8	0	-1.310518	-0.302529	3.667095
70	8	0	-1.972277	-0.270895	0.162243
71	8	0	-1.214234	-3.794875	-3.004127
72	8	0	-4.533667	-0.643013	1.087647
73	8	0	-3.622246	-4.887048	-2.278218
74	8	0	-1.582767	-5.969471	-1.169681
75	8	0	-6.045767	-4.200118	-2.031080
76	8	0	0.469868	-2.920680	1.733170
77	8	0	-8.313435	-3.551617	-1.905640
78	8	0	1.343597	-6.071378	0.846353
79	7	0	1.090878	-2.439838	-1.549323
80	7	0	-0.637479	-6.311695	1.932731
81	6	0	-1.324548	-2.168490	-1.212009
82	6	0	-3.737168	-1.386285	-1.125790
83	6	0	-2.418833	-1.542940	-0.325709
84	6	0	-1.737200	-3.571952	-1.695087
85	6	0	0.049190	-2.165586	-0.497828
86	6	0	-4.765090	-0.518884	-0.324966
87	6	0	-4.208632	-2.752490	-1.521769
88	6	0	-3.267542	-3.760210	-1.876555

89	6	0	-1.217629	-4.729739	-0.819478
90	6	0	-6.232104	-0.935912	-0.446300
91	6	0	0.072454	-3.218993	0.628747
92	6	0	-5.571062	-3.041839	-1.577910
93	6	0	-0.406169	-4.555860	0.252854
94	6	0	-4.606600	0.952356	-0.738136
95	6	0	-6.603404	-2.129033	-1.111268
96	6	0	-7.209146	-0.118048	0.121231
97	6	0	1.599335	-1.147539	-2.107521
98	6	0	2.235951	-3.254430	-1.045268
99	6	0	-7.985609	-2.416362	-1.228036
100	6	0	0.194055	-5.694971	1.007494
101	6	0	-8.569233	-0.438968	0.008343
102	6	0	-8.973180	-1.578742	-0.679267
103	1	0	-1.220205	-1.539383	-2.140460
104	1	0	-3.520818	-0.835290	-2.081545
105	1	0	-2.580809	-2.113353	0.622193
106	1	0	0.191509	-1.161002	-0.004716
107	1	0	-3.577529	1.306603	-0.608327
108	1	0	-5.240755	1.604838	-0.112997
109	1	0	-4.904070	1.124461	-1.777840
110	1	0	-1.806574	0.365747	-0.565425
111	1	0	-0.225377	-3.579468	-3.003443
112	1	0	-3.666501	-0.224992	1.334337
113	1	0	-6.914783	0.784505	0.674878
114	1	0	0.763960	-0.558684	-2.513364
115	1	0	2.149038	-0.521209	-1.385217
116	1	0	2.279456	-1.368854	-2.947066
117	1	0	2.750456	-2.837969	-0.165163

118	1	0	1.894565	-4.278460	-0.789648
119	1	0	2.980122	-3.373162	-1.848863
120	1	0	-2.216764	-5.985390	-1.993775
121	1	0	-5.290918	-4.875656	-2.343601
122	1	0	-9.315399	0.217516	0.462709
123	1	0	-10.026060	-1.822980	-0.781005
124	1	0	-1.576207	-6.004197	2.107704
125	1	0	-0.294535	-7.076063	2.491915
126	1	0	-9.296537	-3.701576	-1.941682

Gaussian 16: ES64L-G16RevA.03 25-Dec-2016

10-May-2021

****

%nprocshared=32

Will use up to 32 processors via shared memory.

%mem=12GB

----

wb97xd/6-311g(2d,p) counterpoise=2

1/38=1,62=2,172=1/1;

2/12=2,17=6,18=5,40=1/2;

1/38=1,53=2,62=2,63=1/22;

3/5=4,6=6,7=102,11=2,25=1,30=1,74=-58/1,2,3;

4//1;

5/5=2,38=5/2;

6/7=2,8=2,9=2,10=2,28=1/1;

1/53=2,62=2,63=1/22;

3/5=7,6=11,7=102,11=2,25=1,30=1,74=-58,82=7/1,2,3;

4//1;

5/5=2,38=5/2;

6/7=2,8=2,9=2,10=2,28=1/1;

1/53=2,62=2,63=1/22(-4);

99/5=1,9=1/99;

----

tetraxicline_GO2_counterpoise

Symbolic Z-matrix:

Charge = 0 Multiplicity = 1 in supermolecule

Charge = 0 Multiplicity = 1 in fragment 1.

Charge = 0 Multiplicity = 1 in fragment 2.

C(Fragment=1) -5.53578 4.62758 0.67635

C(Fragment=1) -4.84664 3.48068 1.11739

C(Fragment=1) -3.44237 3.38093 0.9135

C(Fragment=1) -2.75353 4.43559 0.24705

C(Fragment=1) -3.48119 5.57124 -0.19165

C(Fragment=1) -4.86598 5.65814 0.03428

C(Fragment=1) -2.68519 2.23235 1.36986

C(Fragment=1) -1.32855 4.34689 0.01634

C(Fragment=1) -0.59543 3.21906 0.45571

C(Fragment=1) -1.2947 2.14872 1.14807

C(Fragment=1) 0.80777 3.14513 0.21288

C(Fragment=1) 1.46001 4.20691 -0.46748

C(Fragment=1) 0.71843 5.32227 -0.89163

C(Fragment=1) -0.65532 5.41291 -0.66227

C(Fragment=1) -1.40428 6.56669 -1.10359

C(Fragment=1) -2.74417 6.62669 -0.87021

H(Fragment=1) -0.86839 7.36271 -1.61121

H(Fragment=1)	-6.61021	4.72153	0.85806
H(Fragment=1)	-5.40453	6.54941	-0.30324
H(Fragment=1)	1.22726	6.13903	-1.41017
C(Fragment=1)	-5.55053	2.37699	1.76715
C(Fragment=1)	-4.88362	1.28317	2.22336
C(Fragment=1)	-3.45942	1.18143	2.06012
H(Fragment=1)	-5.39793	0.44614	2.70985
C(Fragment=1)	-2.64391	0.17349	2.43786
C(Fragment=1)	-1.26753	0.10755	2.22074
C(Fragment=1)	-0.48352	1.00145	1.59399
C(Fragment=1)	1.58207	1.99955	0.65521
C(Fragment=1)	0.94734	0.92209	1.35127
C(Fragment=1)	2.9651	1.93148	0.40744
C(Fragment=1)	3.63073	3.01118	-0.29312
C(Fragment=1)	2.90096	4.13403	-0.72437
C(Fragment=1)	3.71886	0.7975	0.84665
C(Fragment=1)	5.03981	2.93011	-0.54047
C(Fragment=1)	5.79484	1.77746	-0.10042
C(Fragment=1)	5.13751	0.70824	0.59097
C(Fragment=1)	7.18066	1.70874	-0.34894
C(Fragment=1)	7.83652	2.79137	-1.03803
C(Fragment=1)	7.12718	3.87719	-1.45343
C(Fragment=1)	5.71245	3.97038	-1.21505
C(Fragment=1)	4.93371	5.09499	-1.6366
C(Fragment=1)	3.57363	5.18133	-1.40418
H(Fragment=1)	3.02715	6.06309	-1.74613
H(Fragment=1)	8.91018	2.71599	-1.21362
H(Fragment=1)	7.61447	4.70265	-1.97557
C(Fragment=1)	1.67793	-0.17245	1.78403

C(Fragment=1)	3.07115	-0.25151	1.53786
H(Fragment=1)	1.19233	-1.00281	2.31818
C(Fragment=1)	3.84019	-1.38858	1.97799
C(Fragment=1)	5.17572	-1.46925	1.73057
C(Fragment=1)	5.86794	-0.42316	1.02069
H(Fragment=1)	3.3064	-2.18764	2.51128
H(Fragment=1)	5.74877	-2.34115	2.06387
C(Fragment=1)	7.90792	0.56569	0.08971
H(Fragment=1)	8.98721	0.5227	-0.09389
C(Fragment=1)	7.2729	-0.47269	0.74166
C(Fragment=1)	8.13962	-1.6094	1.14407
O(Fragment=1)	9.29288	-1.57875	1.50948
O(Fragment=1)	7.50916	-2.82746	1.02118
H(Fragment=1)	8.10754	-3.58694	1.27111
C(Fragment=1)	-7.0258	2.37505	1.9139
O(Fragment=1)	-7.79637	1.47503	1.65301
O(Fragment=1)	-7.51389	3.54676	2.43417
H(Fragment=1)	-8.50482	3.51965	2.56226
O(Fragment=1)	5.48732	6.16006	-2.30191
H(Fragment=1)	6.46737	6.0505	-2.43127
O(Fragment=1)	-3.53452	7.6803	-1.25269
H(Fragment=1)	-3.01308	8.39464	-1.70309
O(Fragment=1)	-1.9624	-0.975	2.90847
O(Fragment=2)	0.4397	-5.13295	-2.67104
O(Fragment=2)	-2.6376	-0.16522	-0.73023
O(Fragment=2)	-2.15102	-5.91134	-2.12582
O(Fragment=2)	-0.38655	-6.79634	-0.4899
O(Fragment=2)	-4.57032	-5.06479	-2.01605
O(Fragment=2)	1.35453	-3.20956	1.96652

O(Fragment=2)	-6.72187	-4.4008	-1.2179
O(Fragment=2)	2.14202	-6.55204	1.98678
N(Fragment=2)	2.59096	-3.66461	-1.17311
N(Fragment=2)	0.00429	-6.42104	2.74615
C(Fragment=2)	0.19944	-3.11999	-1.33034
C(Fragment=2)	-2.06833	-2.25514	-1.8738
C(Fragment=2)	-0.94482	-2.23288	-0.81853
C(Fragment=2)	-0.25856	-4.58204	-1.55485
C(Fragment=2)	1.44674	-3.03989	-0.41709
C(Fragment=2)	-3.11709	-1.11195	-1.70663
C(Fragment=2)	-2.65397	-3.6372	-1.90187
C(Fragment=2)	-1.75577	-4.74619	-1.93483
C(Fragment=2)	0.01567	-5.52527	-0.36529
C(Fragment=2)	-4.487	-1.52192	-1.17267
C(Fragment=2)	1.16133	-3.77174	0.9093
C(Fragment=2)	-4.01928	-3.86679	-1.80739
C(Fragment=2)	0.6483	-5.13874	0.77166
C(Fragment=2)	-4.95448	-2.8453	-1.36163
C(Fragment=2)	-3.33459	-0.39222	-3.0459
C(Fragment=2)	-5.30409	-0.56328	-0.57799
C(Fragment=2)	3.28875	-2.61891	-1.9857
C(Fragment=2)	3.57613	-4.3507	-0.28613
C(Fragment=2)	-6.29014	-3.12935	-0.99161
C(Fragment=2)	1.01991	-6.08912	1.85938
C(Fragment=2)	-6.61372	-0.88353	-0.1868
C(Fragment=2)	-7.12367	-2.15842	-0.40555
H(Fragment=2)	0.50452	-2.73934	-2.34646
H(Fragment=2)	-1.5879	-2.09464	-2.88149
H(Fragment=2)	-0.58689	-1.20129	-0.63737

H(Fragment=2)	-1.32917	-2.57468	0.16277
H(Fragment=2)	1.65012	-1.96322	-0.16655
H(Fragment=2)	-2.39793	-0.02365	-3.4777
H(Fragment=2)	-4.00271	0.47215	-2.93044
H(Fragment=2)	-3.79577	-1.0573	-3.78862
H(Fragment=2)	1.43249	-4.98513	-2.54199
H(Fragment=2)	-1.99502	0.46079	-1.12125
H(Fragment=2)	-4.91107	0.44277	-0.40908
H(Fragment=2)	2.57033	-2.11974	-2.65328
H(Fragment=2)	3.80637	-1.84826	-1.39212
H(Fragment=2)	4.03931	-3.11334	-2.62474
H(Fragment=2)	4.00414	-3.72029	0.50767
H(Fragment=2)	3.11017	-5.23834	0.19015
H(Fragment=2)	4.40802	-4.73655	-0.8969
H(Fragment=2)	-0.88135	-6.97502	-1.38737
H(Fragment=2)	-3.86133	-5.80909	-2.25135
H(Fragment=2)	-7.24033	-0.11789	0.29246
H(Fragment=2)	-8.1439	-2.40128	-0.12239
H(Fragment=2)	-0.9136	-6.01999	2.69191
H(Fragment=2)	0.19053	-7.03495	3.52268
H(Fragment=2)	-7.6594	-4.54262	-0.91463

Stoichiometry C68H40N2O15

Framework group C1[X(C68H40N2O15)]

Deg. of freedom 369

Full point group C1 NOp 1

Largest Abelian subgroup C1 NOp 1

Largest concise Abelian subgroup C1 NOp 1

Standard orientation:

Center Number	Atomic Number	Atomic Type	Coordinates (Angstroms) X	Y	Z
1	6	0	-5.535782	4.627577	0.676353
2	6	0	-4.846638	3.480676	1.117390
3	6	0	-3.442374	3.380934	0.913496
4	6	0	-2.753528	4.435592	0.247046
5	6	0	-3.481192	5.571237	-0.191653
6	6	0	-4.865982	5.658143	0.034280
7	6	0	-2.685187	2.232346	1.369863
8	6	0	-1.328550	4.346892	0.016339
9	6	0	-0.595425	3.219058	0.455705
10	6	0	-1.294695	2.148722	1.148065
11	6	0	0.807768	3.145130	0.212880
12	6	0	1.460006	4.206912	-0.467479
13	6	0	0.718434	5.322273	-0.891625
14	6	0	-0.655316	5.412906	-0.662271
15	6	0	-1.404277	6.566692	-1.103589
16	6	0	-2.744168	6.626690	-0.870209
17	1	0	-0.868394	7.362712	-1.611207
18	1	0	-6.610207	4.721529	0.858059
19	1	0	-5.404529	6.549408	-0.303237
20	1	0	1.227262	6.139031	-1.410173
21	6	0	-5.550534	2.376991	1.767146
22	6	0	-4.883623	1.283170	2.223360
23	6	0	-3.459421	1.181428	2.060117
24	1	0	-5.397933	0.446142	2.709845
25	6	0	-2.643908	0.173485	2.437859

26	6	0	-1.267534	0.107546	2.220736
27	6	0	-0.483517	1.001455	1.593992
28	6	0	1.582067	1.999547	0.655214
29	6	0	0.947338	0.922094	1.351267
30	6	0	2.965099	1.931482	0.407436
31	6	0	3.630729	3.011177	-0.293121
32	6	0	2.900959	4.134035	-0.724368
33	6	0	3.718855	0.797499	0.846652
34	6	0	5.039812	2.930113	-0.540465
35	6	0	5.794842	1.777463	-0.100422
36	6	0	5.137510	0.708240	0.590971
37	6	0	7.180662	1.708741	-0.348940
38	6	0	7.836524	2.791373	-1.038026
39	6	0	7.127175	3.877191	-1.453434
40	6	0	5.712446	3.970378	-1.215051
41	6	0	4.933714	5.094988	-1.636595
42	6	0	3.573631	5.181327	-1.404183
43	1	0	3.027154	6.063088	-1.746130
44	1	0	8.910180	2.715991	-1.213624
45	1	0	7.614473	4.702653	-1.975565
46	6	0	1.677934	-0.172448	1.784033
47	6	0	3.071148	-0.251513	1.537858
48	1	0	1.192328	-1.002811	2.318182
49	6	0	3.840185	-1.388575	1.977992
50	6	0	5.175722	-1.469247	1.730571
51	6	0	5.867938	-0.423158	1.020690
52	1	0	3.306402	-2.187644	2.511285
53	1	0	5.748765	-2.341154	2.063874
54	6	0	7.907921	0.565685	0.089705

55	1	0	8.987214	0.522696	-0.093887
56	6	0	7.272895	-0.472688	0.741656
57	6	0	8.139620	-1.609401	1.144075
58	8	0	9.292879	-1.578745	1.509476
59	8	0	7.509157	-2.827462	1.021183
60	1	0	8.107540	-3.586935	1.271110
61	6	0	-7.025795	2.375045	1.913903
62	8	0	-7.796367	1.475029	1.653005
63	8	0	-7.513890	3.546757	2.434169
64	1	0	-8.504815	3.519653	2.562262
65	8	0	5.487320	6.160059	-2.301910
66	1	0	6.467367	6.050504	-2.431274
67	8	0	-3.534516	7.680296	-1.252691
68	1	0	-3.013076	8.394639	-1.703092
69	8	0	-1.962404	-0.974999	2.908474
70	8	0	0.439696	-5.132955	-2.671041
71	8	0	-2.637600	-0.165219	-0.730230
72	8	0	-2.151024	-5.911336	-2.125824
73	8	0	-0.386548	-6.796336	-0.489903
74	8	0	-4.570323	-5.064793	-2.016045
75	8	0	1.354534	-3.209560	1.966520
76	8	0	-6.721870	-4.400803	-1.217896
77	8	0	2.142015	-6.552040	1.986784
78	7	0	2.590958	-3.664607	-1.173114
79	7	0	0.004291	-6.421040	2.746153
80	6	0	0.199437	-3.119988	-1.330341
81	6	0	-2.068330	-2.255141	-1.873802
82	6	0	-0.944823	-2.232876	-0.818534
83	6	0	-0.258555	-4.582039	-1.554848

84	6	0	1.446739	-3.039887	-0.417091
85	6	0	-3.117086	-1.111953	-1.706628
86	6	0	-2.653968	-3.637198	-1.901874
87	6	0	-1.755772	-4.746193	-1.934825
88	6	0	0.015672	-5.525266	-0.365286
89	6	0	-4.487005	-1.521922	-1.172670
90	6	0	1.161327	-3.771735	0.909296
91	6	0	-4.019278	-3.866788	-1.807389
92	6	0	0.648304	-5.138744	0.771664
93	6	0	-4.954483	-2.845304	-1.361631
94	6	0	-3.334593	-0.392224	-3.045900
95	6	0	-5.304085	-0.563282	-0.577986
96	6	0	3.288748	-2.618908	-1.985698
97	6	0	3.576125	-4.350701	-0.286126
98	6	0	-6.290138	-3.129354	-0.991609
99	6	0	1.019912	-6.089120	1.859377
100	6	0	-6.613720	-0.883527	-0.186802
101	6	0	-7.123673	-2.158423	-0.405545
102	1	0	0.504518	-2.739343	-2.346462
103	1	0	-1.587896	-2.094640	-2.881489
104	1	0	-0.586887	-1.201286	-0.637370
105	1	0	-1.329174	-2.574683	0.162771
106	1	0	1.650117	-1.963223	-0.166551
107	1	0	-2.397934	-0.023653	-3.477703
108	1	0	-4.002710	0.472154	-2.930444
109	1	0	-3.795766	-1.057301	-3.788623
110	1	0	1.432494	-4.985127	-2.541991
111	1	0	-1.995022	0.460789	-1.121246
112	1	0	-4.911067	0.442774	-0.409082

113	1	0	2.570325	-2.119745	-2.653277
114	1	0	3.806374	-1.848258	-1.392119
115	1	0	4.039306	-3.113341	-2.624735
116	1	0	4.004139	-3.720286	0.507667
117	1	0	3.110173	-5.238344	0.190147
118	1	0	4.408016	-4.736548	-0.896896
119	1	0	-0.881346	-6.975016	-1.387365
120	1	0	-3.861333	-5.809093	-2.251345
121	1	0	-7.240333	-0.117889	0.292463
122	1	0	-8.143903	-2.401280	-0.122385
123	1	0	-0.913604	-6.019994	2.691911
124	1	0	0.190529	-7.034952	3.522684
125	1	0	-7.659397	-4.542615	-0.914625

Gaussian 16: ES64L-G16RevA.03 25-Dec-2016

9-May-2021

****

%chk=TETRACICLINA_QUITOSANE_wb97xd_2.chk

%nprocshared=32

Will use up to   32 processors via shared memory.

%mem=15GB

----

wb97xd/6-311g(2d,p) counterpoise=2

1/38=1,62=2,172=1/1;

2/12=2,17=6,18=5,40=1/2;

1/38=1,53=2,62=2,63=1/22;

3/5=4,6=6,7=102,11=2,25=1,30=1,74=-58/1,2,3;

4//1;

5/5=2,38=5/2;

6/7=2,8=2,9=2,10=2,28=1/1;

1/53=2,62=2,63=1/22;

3/5=7,6=11,7=102,11=2,25=1,30=1,74=-58,82=7/1,2,3;

4//1;

5/5=2,38=5/2;

6/7=2,8=2,9=2,10=2,28=1/1;

1/53=2,62=2,63=1/22(-4);

99/5=1,9=1/99;

----

tetraciclina_quitosano_CP

Symbolic Z-matrix:

Charge = 0 Multiplicity = 1 in supermolecule

Charge = 0 Multiplicity = 1 in fragment 1.

Charge = 0 Multiplicity = 1 in fragment 2.

C(Fragment=2) 1.8026 -2.12165 3.45552

C(Fragment=2) 1.79167 -0.87555 2.57799

C(Fragment=2) 2.55965 -1.20268 1.30494

C(Fragment=2) 1.98231 -3.50168 1.39404

C(Fragment=2) 1.22763 -3.30736 2.7038

H(Fragment=2) 0.75389 -0.66513 2.30355

H(Fragment=2) 2.84751 -2.36127 3.71926

H(Fragment=2) 3.02477 -3.77835 1.6075

H(Fragment=2) 0.17494 -3.09805 2.471

H(Fragment=2) 3.60871 -1.4342 1.54482

C(Fragment=2) 3.01958 0.51328 -1.7743

C(Fragment=2) 4.82044 1.98772 -2.21987

C(Fragment=2)	5.53336	1.58185	-0.92672
C(Fragment=2)	4.5146	1.14753	0.11905
C(Fragment=2)	3.60404	0.07299	-0.43635
H(Fragment=2)	2.34027	1.35998	-1.59102
H(Fragment=2)	6.16579	0.71646	-1.16151
H(Fragment=2)	3.87407	2.01219	0.36629
H(Fragment=2)	4.15905	-0.86383	-0.55743
H(Fragment=2)	4.18784	2.87081	-2.04352
O(Fragment=2)	1.97106	-2.29057	0.63509
O(Fragment=2)	2.50803	-0.11572	0.43808
O(Fragment=2)	4.03142	0.91028	-2.6799
O(Fragment=2)	5.70014	2.34328	-3.22546
H(Fragment=2)	6.18954	1.5545	-3.47743
O(Fragment=2)	5.15024	0.67502	1.2811
H(Fragment=2)	5.91289	1.2539	1.40838
C(Fragment=2)	1.34583	-4.55196	0.50557
H(Fragment=2)	0.27744	-4.3239	0.40814
H(Fragment=2)	1.44648	-5.52846	0.97709
C(Fragment=2)	2.22637	-0.58825	-2.44096
H(Fragment=2)	2.92254	-1.36218	-2.79063
H(Fragment=2)	1.55582	-1.03887	-1.70895
O(Fragment=2)	1.42796	-0.09574	-3.49003
H(Fragment=2)	2.00659	0.326	-4.13
O(Fragment=2)	1.98027	-4.61134	-0.75576
H(Fragment=2)	2.09434	-3.70004	-1.0394
O(Fragment=2)	1.35567	-4.49949	3.44552
H(Fragment=2)	0.96474	-4.33103	4.30665
O(Fragment=2)	1.0485	-1.91746	4.62287
H(Fragment=2)	1.27471	-1.0257	4.9155

N(Fragment=2)	2.29896	0.25942	3.33104
H(Fragment=2)	1.94754	1.11842	2.9233
H(Fragment=2)	3.3124	0.29811	3.27613
N(Fragment=2)	6.38007	2.60284	-0.33196
H(Fragment=2)	7.14791	2.82721	-0.953
H(Fragment=2)	5.85257	3.46162	-0.2049
O(Fragment=1)	-2.79897	-0.65323	-3.25676
O(Fragment=1)	0.93531	2.03971	1.15014
O(Fragment=1)	-3.9302	1.67465	-2.46591
O(Fragment=1)	-5.00234	-0.49331	-1.4346
O(Fragment=1)	-3.87073	3.6642	-0.9512
O(Fragment=1)	-1.90289	-2.99166	1.05041
O(Fragment=1)	-4.31966	4.92695	1.21495
O(Fragment=1)	-5.8796	-2.52414	0.12492
N(Fragment=1)	-1.54722	-2.93444	-2.25719
N(Fragment=1)	-4.49603	-2.7809	1.89308
C(Fragment=1)	-1.27271	-0.63346	-1.36926
C(Fragment=1)	-0.74312	1.64694	-0.60346
C(Fragment=1)	-0.83091	0.19305	-0.15797
C(Fragment=1)	-2.65024	-0.24814	-1.91622
C(Fragment=1)	-1.24706	-2.12831	-1.05779
C(Fragment=1)	-0.05842	2.65552	0.35255
C(Fragment=1)	-2.10776	2.10968	-1.04425
C(Fragment=1)	-2.90617	1.27294	-1.87918
C(Fragment=1)	-3.80083	-0.90456	-1.11676
C(Fragment=1)	-1.03031	3.33899	1.29427
C(Fragment=1)	-2.2573	-2.36999	0.058
C(Fragment=1)	-2.74834	3.19395	-0.46935
C(Fragment=1)	-3.60315	-1.85663	-0.15701

C(Fragment=1)	-2.28678	3.74093	0.81133
C(Fragment=1)	0.60585	3.75855	-0.48944
C(Fragment=1)	-0.62359	3.66842	2.57404
C(Fragment=1)	-0.37131	-3.02032	-3.12019
C(Fragment=1)	-2.01349	-4.2802	-1.93575
C(Fragment=1)	-3.11132	4.51992	1.64709
C(Fragment=1)	-4.7737	-2.42138	0.60846
C(Fragment=1)	-1.45534	4.41861	3.39005
C(Fragment=1)	-2.68074	4.85026	2.92958
H(Fragment=1)	-0.57632	-0.42063	-2.18407
H(Fragment=1)	-0.10679	1.64174	-1.49965
H(Fragment=1)	0.13575	-0.19034	0.16433
H(Fragment=1)	-1.52219	0.09132	0.68386
H(Fragment=1)	-0.26869	-2.39864	-0.63928
H(Fragment=1)	1.37854	3.31975	-1.12705
H(Fragment=1)	1.06847	4.49586	0.16766
H(Fragment=1)	-0.11901	4.26355	-1.13111
H(Fragment=1)	-2.58119	-1.60535	-3.23421
H(Fragment=1)	1.40192	1.34794	0.66388
H(Fragment=1)	0.34461	3.34376	2.92394
H(Fragment=1)	-0.03066	-2.02892	-3.41764
H(Fragment=1)	0.45079	-3.55802	-2.62416
H(Fragment=1)	-0.63523	-3.56613	-4.02743
H(Fragment=1)	-1.27989	-4.84546	-1.34353
H(Fragment=1)	-2.95615	-4.23938	-1.39044
H(Fragment=1)	-2.19184	-4.81812	-2.86747
H(Fragment=1)	-4.92068	0.25191	-2.06458
H(Fragment=1)	-4.11155	3.08541	-1.72336
H(Fragment=1)	-1.14016	4.67629	4.39413

H(Fragment=1) -3.32853 5.44392 3.5667

H(Fragment=1) -3.52962 -2.96471 2.12611

H(Fragment=1) -5.21631 -3.32158 2.34369

H(Fragment=1) -4.75982 5.39362 1.92887

Stoichiometry C34H48N4O17

Framework group C1[X(C34H48N4O17)]

Deg. of freedom 303

Full point group C1 NOp 1

Largest Abelian subgroup C1 NOp 1

Largest concise Abelian subgroup C1 NOp 1

Standard orientation:

Center Number	Atomic Number	Atomic Type	Coordinates (Angstroms) X	Y	Z
1	6	0	1.802598	-2.121648	3.455520
2	6	0	1.791669	-0.875548	2.577990
3	6	0	2.559650	-1.202677	1.304935
4	6	0	1.982313	-3.501676	1.394037
5	6	0	1.227631	-3.307360	2.703796
6	1	0	0.753892	-0.665130	2.303550
7	1	0	2.847508	-2.361270	3.719259
8	1	0	3.024775	-3.778354	1.607500
9	1	0	0.174944	-3.098054	2.471002
10	1	0	3.608710	-1.434200	1.544824
11	6	0	3.019577	0.513277	-1.774304
12	6	0	4.820441	1.987717	-2.219872
13	6	0	5.533361	1.581850	-0.926724

14	6	0	4.514603	1.147530	0.119046
15	6	0	3.604042	0.072987	-0.436350
16	1	0	2.340273	1.359984	-1.591016
17	1	0	6.165792	0.716455	-1.161514
18	1	0	3.874065	2.012190	0.366286
19	1	0	4.159054	-0.863827	-0.557427
20	1	0	4.187842	2.870809	-2.043521
21	8	0	1.971060	-2.290567	0.635089
22	8	0	2.508025	-0.115719	0.438081
23	8	0	4.031418	0.910280	-2.679901
24	8	0	5.700136	2.343281	-3.225461
25	1	0	6.189540	1.554501	-3.477427
26	8	0	5.150235	0.675018	1.281099
27	1	0	5.912886	1.253903	1.408376
28	6	0	1.345828	-4.551963	0.505573
29	1	0	0.277444	-4.323899	0.408141
30	1	0	1.446476	-5.528464	0.977093
31	6	0	2.226371	-0.588249	-2.440963
32	1	0	2.922536	-1.362183	-2.790633
33	1	0	1.555820	-1.038866	-1.708947
34	8	0	1.427964	-0.095743	-3.490028
35	1	0	2.006587	0.326000	-4.130003
36	8	0	1.980270	-4.611342	-0.755759
37	1	0	2.094337	-3.700043	-1.039401
38	8	0	1.355669	-4.499493	3.445518
39	1	0	0.964735	-4.331030	4.306648
40	8	0	1.048503	-1.917456	4.622866
41	1	0	1.274712	-1.025697	4.915495
42	7	0	2.298960	0.259418	3.331037

43	1	0	1.947542	1.118416	2.923301
44	1	0	3.312399	0.298106	3.276128
45	7	0	6.380068	2.602838	-0.331959
46	1	0	7.147914	2.827208	-0.953001
47	1	0	5.852565	3.461617	-0.204901
48	8	0	-2.798969	-0.653225	-3.256761
49	8	0	0.935307	2.039711	1.150144
50	8	0	-3.930201	1.674652	-2.465909
51	8	0	-5.002342	-0.493305	-1.434602
52	8	0	-3.870732	3.664201	-0.951200
53	8	0	-1.902893	-2.991659	1.050412
54	8	0	-4.319661	4.926950	1.214951
55	8	0	-5.879601	-2.524144	0.124922
56	7	0	-1.547216	-2.934441	-2.257192
57	7	0	-4.496026	-2.780898	1.893081
58	6	0	-1.272710	-0.633458	-1.369257
59	6	0	-0.743120	1.646938	-0.603455
60	6	0	-0.830912	0.193049	-0.157969
61	6	0	-2.650243	-0.248135	-1.916217
62	6	0	-1.247056	-2.128309	-1.057788
63	6	0	-0.058416	2.655520	0.352547
64	6	0	-2.107755	2.109676	-1.044250
65	6	0	-2.906167	1.272939	-1.879183
66	6	0	-3.800830	-0.904555	-1.116756
67	6	0	-1.030314	3.338987	1.294273
68	6	0	-2.257297	-2.369992	0.058004
69	6	0	-2.748339	3.193949	-0.469351
70	6	0	-3.603145	-1.856633	-0.157013
71	6	0	-2.286783	3.740934	0.811334

72	6	0	0.605849	3.758553	-0.489441
73	6	0	-0.623586	3.668424	2.574039
74	6	0	-0.371305	-3.020317	-3.120193
75	6	0	-2.013487	-4.280201	-1.935752
76	6	0	-3.111325	4.519922	1.647085
77	6	0	-4.773701	-2.421383	0.608455
78	6	0	-1.455343	4.418605	3.390045
79	6	0	-2.680743	4.850260	2.929583
80	1	0	-0.576316	-0.420629	-2.184066
81	1	0	-0.106785	1.641742	-1.499652
82	1	0	0.135752	-0.190343	0.164331
83	1	0	-1.522188	0.091317	0.683862
84	1	0	-0.268690	-2.398636	-0.639278
85	1	0	1.378543	3.319750	-1.127046
86	1	0	1.068469	4.495857	0.167662
87	1	0	-0.119007	4.263554	-1.131112
88	1	0	-2.581186	-1.605351	-3.234206
89	1	0	1.401915	1.347940	0.663875
90	1	0	0.344608	3.343758	2.923936
91	1	0	-0.030664	-2.028917	-3.417641
92	1	0	0.450792	-3.558016	-2.624162
93	1	0	-0.635229	-3.566134	-4.027430
94	1	0	-1.279888	-4.845457	-1.343533
95	1	0	-2.956147	-4.239379	-1.390440
96	1	0	-2.191840	-4.818117	-2.867470
97	1	0	-4.920676	0.251912	-2.064576
98	1	0	-4.111549	3.085407	-1.723364
99	1	0	-1.140156	4.676288	4.394132
100	1	0	-3.328527	5.443924	3.566698

101	1	0	-3.529620	-2.964713	2.126110
102	1	0	-5.216305	-3.321575	2.343685
103	1	0	-4.759815	5.393620	1.928867
